# P62 promotes FSH-induced antral follicle formation by directing degradation of ubiquitinated WT1

**DOI:** 10.1007/s00018-024-05251-x

**Published:** 2024-05-20

**Authors:** Ting Zhao, Meina He, Zijian Zhu, Tuo Zhang, Wenying Zheng, Shaogang Qin, Meng Gao, Wenji Wang, Ziqi Chen, Jun Han, Longping Liu, Bo Zhou, Haibin Wang, Hua Zhang, Guoliang Xia, Jianbin Wang, Fengchao Wang, Chao Wang

**Affiliations:** 1 State Key Laboratory of Animal Biotech Breeding, College of Biological Sciences, China Agricultural University, Beijing, 100193 China; 2 College of Basic Medicine, Guizhou Medical University, Guiyang, Guizhou Province 550025 China; 3 Guizhou Provincial Key Laboratory of Pathogenesis and Drug Research on Common Chronic Diseases, Department of Physiology, College of Basic Medicine, Guizhou Medical University, Guiyang, Guizhou Province 550025 China; 4 School of Life Science, Taizhou University, Taizhou, 318000 China; 5 School of Life Sciences, Tsinghua University, Beijing, 100084 China; 6 Fujian Provincial Key Laboratory of Reproductive Health Research, Department of Obstetrics and Gynecology, School of Medicine, The First Affiliated Hospital of Xiamen University, Xiamen University, Xiamen, Fujian Province 361005 China; 7 Key Laboratory of Ministry of Education for Conservation and Utilization of Special Biological Resources in the Western China, College of Life Science, Ningxia University, Yinchuan, 750021 China; 8 Transgenic Animal Center, National Institute of Biological Sciences, Beijing, 102206 China; 9 China Agricultural University, No.2 Yuan Ming Yuan West Road, Haidian District, Beijing, 100193 China

**Keywords:** p62, FSH, Ubiquitinatized WT1, Granulosa cell differentiation, Antral follicles formation

## Abstract

In females, the pathophysiological mechanism of poor ovarian response (POR) is not fully understood. Considering the expression level of p62 was significantly reduced in the granulosa cells (GCs) of POR patients, this study focused on identifying the role of the selective autophagy receptor p62 in conducting the effect of follicle-stimulating hormone (FSH) on antral follicles (AFs) formation in female mice. The results showed that p62 in GCs was FSH responsive and that its level increased to a peak and then decreased time-dependently either in ovaries or in GCs after gonadotropin induction in vivo. GC-specific deletion of *p62* resulted in subfertility, a significantly reduced number of AFs and irregular estrous cycles, which were same as pathophysiological symptom of POR. By conducting mass spectrum analysis, we found the ubiquitination of proteins was decreased, and autophagic flux was blocked in GCs. Specifically, the level of nonubiquitinated Wilms tumor 1 homolog (WT1), a transcription factor and negative controller of GC differentiation, increased steadily. Co-IP results showed that *p62* deletion increased the level of ubiquitin-specific peptidase 5 (USP5), which blocked the ubiquitination of WT1. Furthermore, a joint analysis of RNA-seq and the spatial transcriptome sequencing data showed the expression of steroid metabolic genes and FSH receptors pivotal for GCs differentiation decreased unanimously. Accordingly, the accumulation of WT1 in GCs deficient of *p62* decreased steroid hormone levels and reduced FSH responsiveness, while the availability of p62 in GCs simultaneously ensured the degradation of WT1 through the ubiquitin‒proteasome system and autophagolysosomal system. Therefore, p62 in GCs participates in GC differentiation and AF formation in FSH induction by dynamically controlling the degradation of WT1. The findings of the study contributes to further study the pathology of POR.

**Supplementary Information:**

The online version contains supplementary material available at 10.1007/s00018-024-05251-x.

## Introduction

Poor ovarian response (POR) refers to the state of poor ovarian response to gonadotropins (Gn) stimulation, which is mainly manifested as fewer follicles developed during ovarian stimulation cycle, low estrogen content in serum, low number of oocytes obtained and low clinical pregnancy rate [1]. POR accounts for 9–24% of all infertile women, and in elderly infertile women, the proportion of ovarian adverse reaction is as high as 54–62% [2]. In IVF assisted pregnancy treatment, elderly POR patients may decrease the number of oocytes obtained, decrease the rate of fine embryo, increase the cycle cancellation rate, increase the risk of embryo aneuploidy, and significantly reduce the clinical pregnancy rate and cumulative live birth rate, thus increasing the difficulty of assisted pregnancy [3]. Studies have shown that decreased gonadotropin secretion, oxidative stress, decreased FSHR expression and mitochondrial function are related to the occurrence of POR. The pathophysiological mechanism of POR is not fully understood [4–7].

Mammalian ovarian follicle cyclic recruitment and dominant follicle selection is a complex and dynamic physiological process that requires the interaction of different hormones and cell signaling pathways [8–10]. However, not all cyclically recruited follicles develop into fully grown follicles and ovulate; instead, most of them degenerate and become atretic during antral follicle (AF) formation for complex reasons [11, 12]. Follicle-stimulating hormone (FSH) is one of the most important hormones affecting the fate of cyclically recruited follicles [13, 14]. FSH promotes follicular development through binding to the FSH receptor (FSHR) residing on the membrane of granulosa cells (GCs) to increase GC proliferation and differentiation and the synthesis of steroid hormones such as sex hormones and peptides [15–19]. Consequently, a lack of sufficient responsiveness to FSH is believed to be the key reason for induction of follicle atresia, although the detailed mechanisms need further exploration [13, 20]. Recent studies have reported that apoptosis and autophagy, the two most important programmed cell death patterns, are involved in follicle growth and atresia, GC differentiation, and the reproductive cycle [21–25]. Rapidly proliferated and differentiated GCs provide nutrients, paracrine hormones and natriuretic peptides to support oocyte growth and maturity, assisting in the formation of antral cells and regulating meiosis before the LH surge occurs [26–28]. Interestingly, during AF formation, FSH promotes the activation of autophagy in GCs by upregulating hypoxia-inducible factor-1α (HIF-1α) and downregulating mammalian target of rapamycin (mTOR) in mice [29–32]. Although autophagy gene expression is detectable throughout the ovaries [33, 34], autolysosome have been observed more frequently in the GC layers of atretic follicles [35]. In atretic follicles, GC apoptosis is induced by overactive autophagy through elevations in reactive oxygen species production and oxidized low-density lipoprotein receptor expression [36, 37]. Hence, autophagy in GCs during FSH-induced follicular dominance selection is important for maintaining follicle development, although the underlying mechanisms need exploration.

Interestingly, recent studies have shown that the autophagy activity of GCs in growing follicles regulates the expression level of Wilms tumor 1 homolog (WT1), a pivotal zinc finger nuclear transcription factor in reproduction [23, 38]. Cumulative studies have shown that the expression of FSHR, 3β-hydroxysteroid dehydrogenase (3β-HSD), and aromatase are all targets of WT1 [39–42]. Importantly, inactivation of WT1 results in pregranulosa cell (pre-GC)-to-steroidogenic cell transformation, which in turn causes defects in ovary development, while mutation of *Wt1* results in both upregulation of these genes and premature differentiation of GCs [41]. Despite these findings, how GC differentiation-related molecules, including WT1, are finely tuned in response to FSH to facilitate AF formation and promote follicle selection has not been fully elucidated.

Protein ubiquitination affects protein degradation and autophagy. Studies have shown that the ubiquitination-deubiquitylation system regulates female reproductive processes, including the cell cycle and oocyte maturation, mainly by regulating protein stability [43–45]. Interestingly, FSH-regulated protein homeostasis in GCs through ubiquitin-proteasome degradation pathways, including recombinant human cyclin-D2 (CCND2), breast cancer 1 (BRCA1), androgen receptor (AR), etc., is also important to improve follicle development [46–48]. p62/SQSTM1 (sequestosome 1), the selective autophagy receptor, participates in the activities of both the ubiquitin-proteasome system (UPS) and autolysosome system (ALS) [49]. The protein degradation processes in a cell depends on the dual functions of p62. As an ubiquitin and LC3 binding protein, p62 promotes the formation and degradation of ubiquitination aggregates through its ubiquitin-binding domains by acting as a scaffold protein [50]. In contrast, deubiquitinating enzymes, such as USP-related proteins, reverse the effects of ubiquitination by removing the ubiquitin chain from the target protein [51]. Recently, we demonstrated that during primordial follicle formation in perinatal mouse ovaries, lysine-specific demethylase 1 (LSD1) regulates *p62* transcription through its histone demethylase activity, which in turn protects oocytes from autophagy [52]. Given that p62 is also expressed in ovarian somatic cells after birth [38, 53], we wonder whether p62 in GCs is essential for follicular development and whether the action of p62 is involved in promoting follicle autophagy and improving AF formation.

According to this study, we found decreased *p62* expression in GCs of POR patients, suggesting that p62 is one of the genetic causes associated with POR. Based on the mouse models, it was found that *p62* deletion in GCs decreased the responsiveness of GCs to FSH, resulting in a decrease in the number of AFs and the fertility of mice. P62 in the GCs of cyclically recruited follicles is vital for regulating cell differentiation during AFs formation. Specifically, p62, as one of the important downstream effectors of FSH, promotes GC differentiation by mediating WT1 ubiquitination and degradation, which eventually affects follicle fate. p62 is considered a candidate of post-translational modification for POR diagnosis and pathological studies.

## Results

### Low p62 was related to POR in women and p62 in GCs is an FSH-responsive protein during the antral follicle formation

**Fig. 1:**
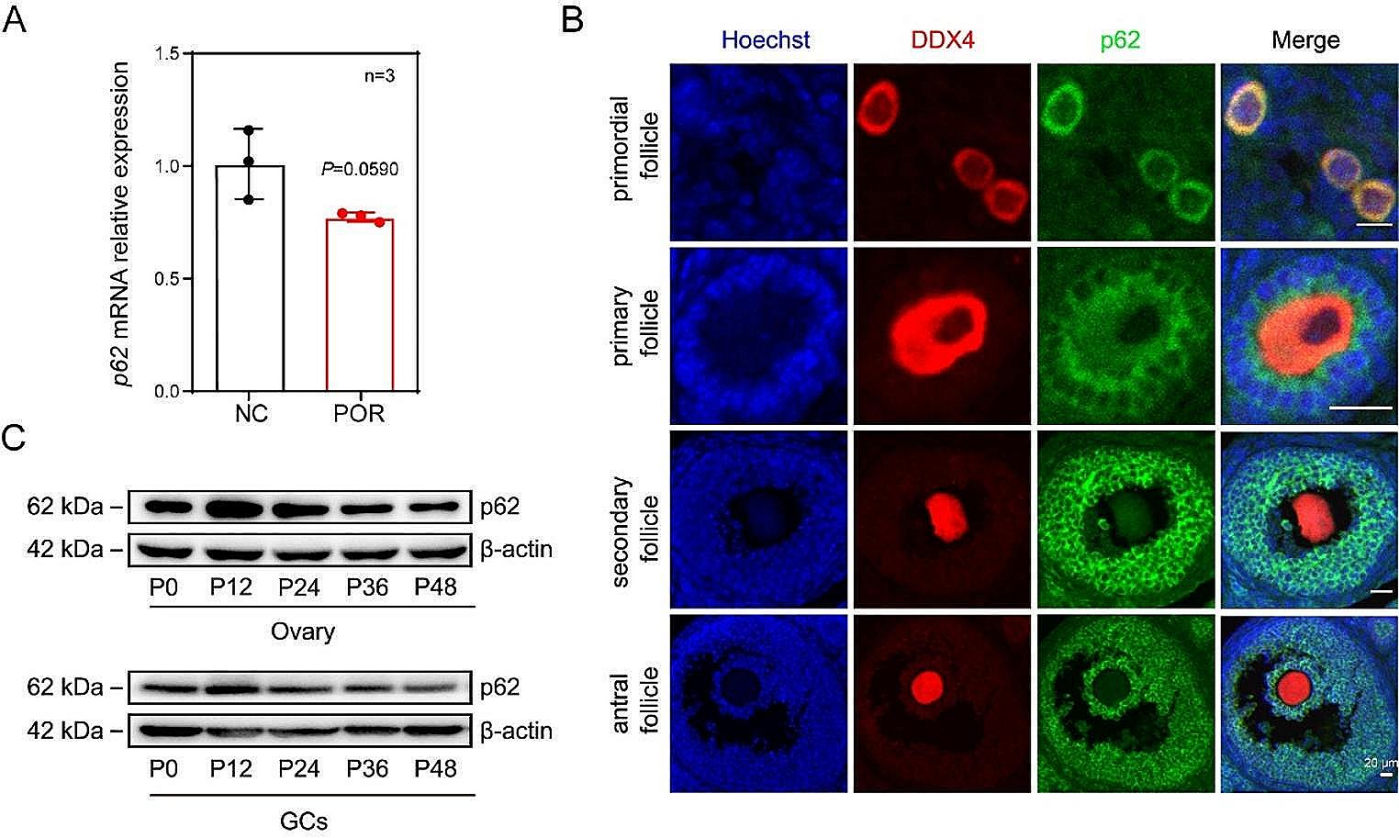
Low p62 was related to POR in women and p62 in GCs is one of the key downstream proteins in response to FSH. (A) PCR results showed the levels of p62 in the GCs of NC and POR female patients, revealing low *p62* in GCs of POR (*n* = 3). (B) Immunofluorescence staining of p62 (green), DDX4 (red) and Hoechst (blue) in the ovaries of PD35 mice. Scale bar: 50 μm. (C) The three-week-old mice were injected with PMSG. The expression pattern of p62 in the whole ovary and the isolated GCs were determined by western blotting at 0 h, 12 h, 24 h, 36 h and 48 h after induction, respectively (*n* = 3)

To determine the genetic cause of POR, we systematically analyzed the basic information of female patients with POR. Interestingly, compared to the normal group, we noted a decrease in *p62* mRNA expression levels in patients with POR, which has not been reported before (Fig. 1A). These findings suggest that p62 may be a candidate for POR.

To identify the potential function of p62 in female reproduction, we first examined the location and expression pattern of p62 in the cells of follicles at all developmental stages in mice 35 days post-partum (PD35). The results showed that p62 was generally expressed in both the cytoplasm of GCs and oocytes in the follicles. Specifically, the levels of p62 in oocytes remained stable in primordial follicles (PmFs) and primary follicles (PFs). The levels of p62 in GCs of both secondary follicles (SFs) and antral follicles (AFs) remained at relatively higher levels than those found in the PmFs and PFs (Fig. 1B), implying that p62 may be more important for supporting SF growth.

To identify whether the stronger expression of p62 in GCs of SFs is responsive to FSH induction, three-week-old mice were injected with pregnant mare serum gonadotropin (PMSG) for 48 h to induce rapid follicle growth. The expression levels of p62 in the whole ovary and isolated GCs were examined at P0, P12, P24, P36 and P48 after PMSG treatment. The results showed that compared to that at P0, the level of p62 protein in either the whole ovaries or in the isolated GCs increased in a time-dependent manner, peaked at P12, and then decreased gradually over time (Fig. 1C). The findings indicate that the expression of p62 is FSH responsive and may play an important role in the growth and differentiation of GCs during follicular development.

### p62 in GCs was required to support antral follicular formation

**Fig. 2:**
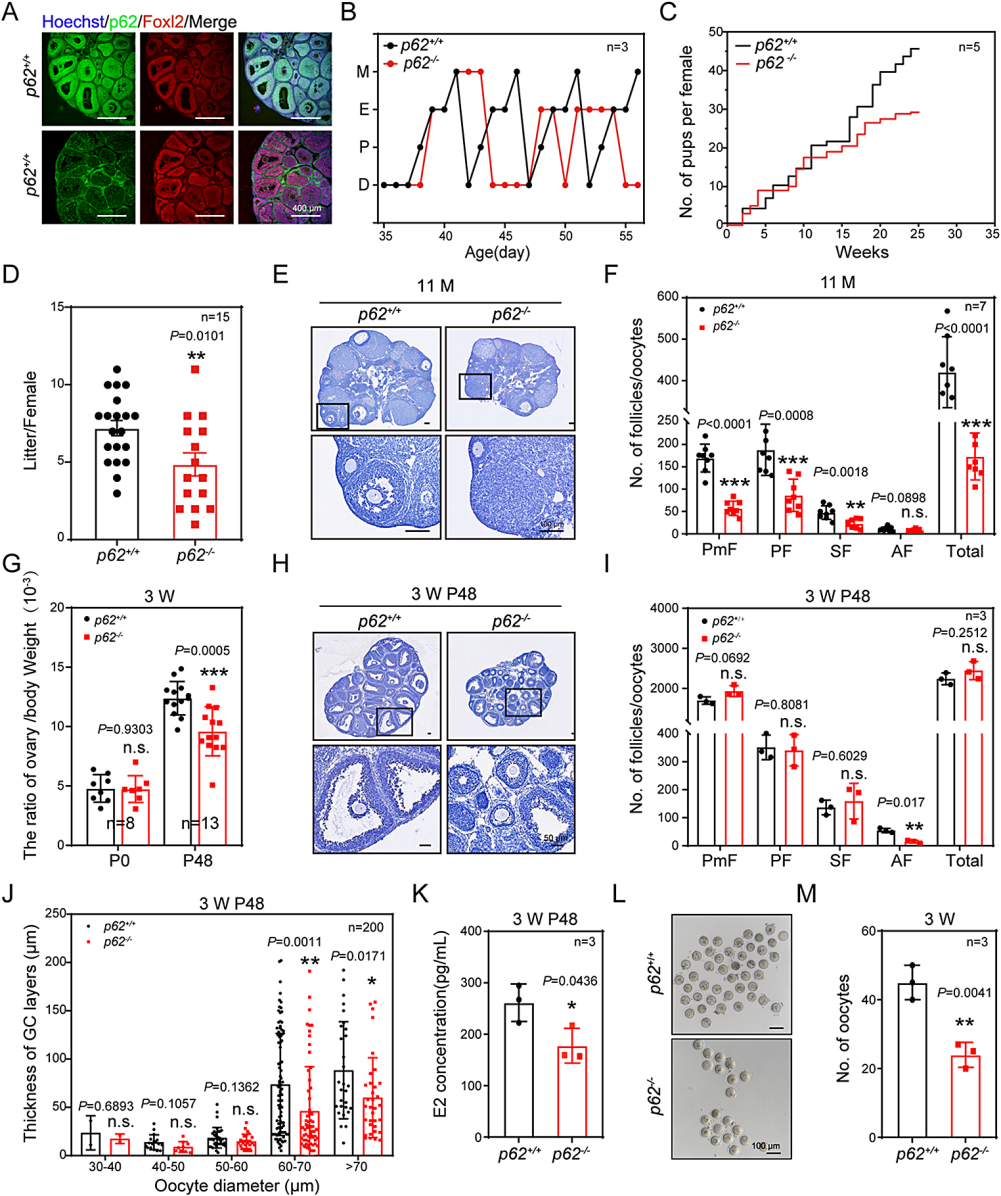
p62 in GCs was required to support AF formation. (**A**) Immunofluorescence staining of the p62 (green), FOXL2 (red) and Hoechst (blue) in three-week-old mouse ovaries 48 h after PMSG treatment. Scale bar: 400 μm. (**B**) Detection of the estrous cycle in *p62*^*+/+*^ and *p62*^*−/−*^ adult mice (*n* = 3). (**C**) Breeding test (*n* = 5). (**D**) Average litter size of both *p62*^*+/+*^ and *p62*^*−/−*^ females (*n* = 15). (**E**) Representative histology of ovarian sections from 11-month-old *p62*^*−/−*^ females stained with hematoxylin. The boxed regions in the panels are magnified. Scale bar: 100 μm. (**F**) The number of follicles at different developmental stages were determined for 11-month-old females (*n* = 7). (**G**) The ovarian weight to body weight ratio at the time points of P0 (*n* = 8) and P48 (*n* = 13), respectively. (**H**) Hematoxylin-stained ovary sections of P48 mice. The boxed region of each figure was magnified accordingly. Scale bar: 50 μm. (**I**) Statistics of the numbers of PmFs, PFs, SFs, AFs and TFs in ovaries at P48 (*n* = 3). (**J**) The thicknesses of GC layers in follicles of P48 ovaries at all developmental stages were compared based on oocyte diameter (*n* = 200). (**K**) Levels of serum E2 after mice were treated with PMSG for 48 h (*n* = 4). (**L**) Oocyte morphology after superovulation. Scale bar: 100 μm. (**M**) Statistical data of L (*n* = 3). PmF, primordial follicle; PF, primary follicle; SF, secondary follicle; AF, antral follicle; TF: total follicle

To identify the exact roles of p62 in the GCs of growing follicles, p62 was specifically deleted in pre-GCs using *Foxl2-Cre* mice. The knockout mice, *p62*^*flox/flox*^; *Foxl2-Cre*, were marked by *p62*^*−/−*^ and the wild-type mice, *p62*^*flox/flox*^, were marked by *p62*^*+/+*^ (Fig. S1A, B). The knockout efficiency of *p62*^*−/−*^ mice was examined by immunofluorescence (Fig. 2A) and confirmed by western blotting and real time PCR (RT-PCR) using ovarian tissues (Fig. S1C).

First, to explore the effect of *p62* deletion on female fertility, a breeding assay was carried out wherein the control or *p62*^*−/−*^ mice were mated with wild-type males of proven fertility for 6 months. The results showed that the fertility of *p62*^*−/−*^ females was impaired in a time-dependently manner and tended to decrease after 25 weeks (Fig. 2C). The average litter size of *p62*^*−/−*^ females was significantly reduced compared with that of *p62*^*+/+*^ mice (Fig. 2D). Next, the estrous cycle of the *p62*^*−/−*^ mice was examined (Fig. S1D). The results showed that the estrous cycle of *p62*^*−/−*^ mice was irregular, with either incomplete cycles or unstable duration of the estrus stage between the cycles (Fig. 2B). Finally, our observation of the sections of 11-months-old mouse ovaries using hematoxylin and eosin (H&E) staining revealed that the number of follicles at all developmental stages of the *p62*^*−/−*^ mice were significantly decreased (Fig. 2E, F). Analyses of the gonadal hormones FSH, LH, and E2 in 11-month-old *p62*^*−/−*^ mice showed that both LH and E2 levels were significantly lower than those in *p62*^*+/+*^ mice (Fig. S1E, S1G), but the FSH content was not different between *p62*^*−/−*^ mice and *p62*^*+/+*^ mice (Fig. S1F). However, the LH: FSH ratio showed a significant reduction (Fig. S1H). These results indicated that p62 in GCs deeply affected the female fertility and follicular development.

To clarify how *p62* deficiency hindered mouse fertility, we evaluated the number of follicles at all developmental stages in the ovaries of P0 and P48. The results showed that the morphology (Fig. S2A), size (Fig. S2B) and number of follicles (Fig. S2C) at different developmental stages of P0 *p62*^*−/−*^ females were similar to those of the control. At P48, the ovarian size and ovary weight to body weight ratio were both significantly lower in *p62*^*−/−*^ mice, than in *p62*^*+/+*^ mice (Fig. 2G, H). More importantly, both the number of AFs (Fig. 2I), and the ratio of AFs/SFs (Fig. S2F) were significantly lower in *p62*^*−/−*^ mice than in the control. Meanwhile, the number of atretic follicles in the *p62*^*−/−*^ mice were significantly greater than those in the control mice (Fig. S2G, 2 H).

Next, to evaluate the specific effect of deletion of *p62* on the development of follicles, we analyzed the size of follicles and the thickness of GCs layers in follicles that had the same oocyte diameter between the *p62*^*+/+*^ and the *p62*^*−/−*^ females in 3-week-old mice. The number of GC layers was significantly lower in *p62*^*−/−*^ mice than in *p62*^*+/+*^ mice at P48, although the oocyte diameters in both groups were greater than 60 μm (Fig. 2J). Specifically, the difference of GCs layers occurred during SFs transforming into AFs (Fig. S2E). We then found that the levels of E2 decreased significantly at P48 in the *p62*^*−/−*^ mice (Fig. 2K). Furthermore, the superovulation treatment results showed that the ovulation number in the *p62*^*−/−*^ mice was significantly lower than that in the *p62*^*+/+*^ mice (Fig. 2L, M). Together, the findings indicate that loss of p62 function in GCs leads to the developmental failure of AF formation from SFs.

### The cell fate of the GCs were disturbed after deletion of ***p62***

**Fig. 3:**
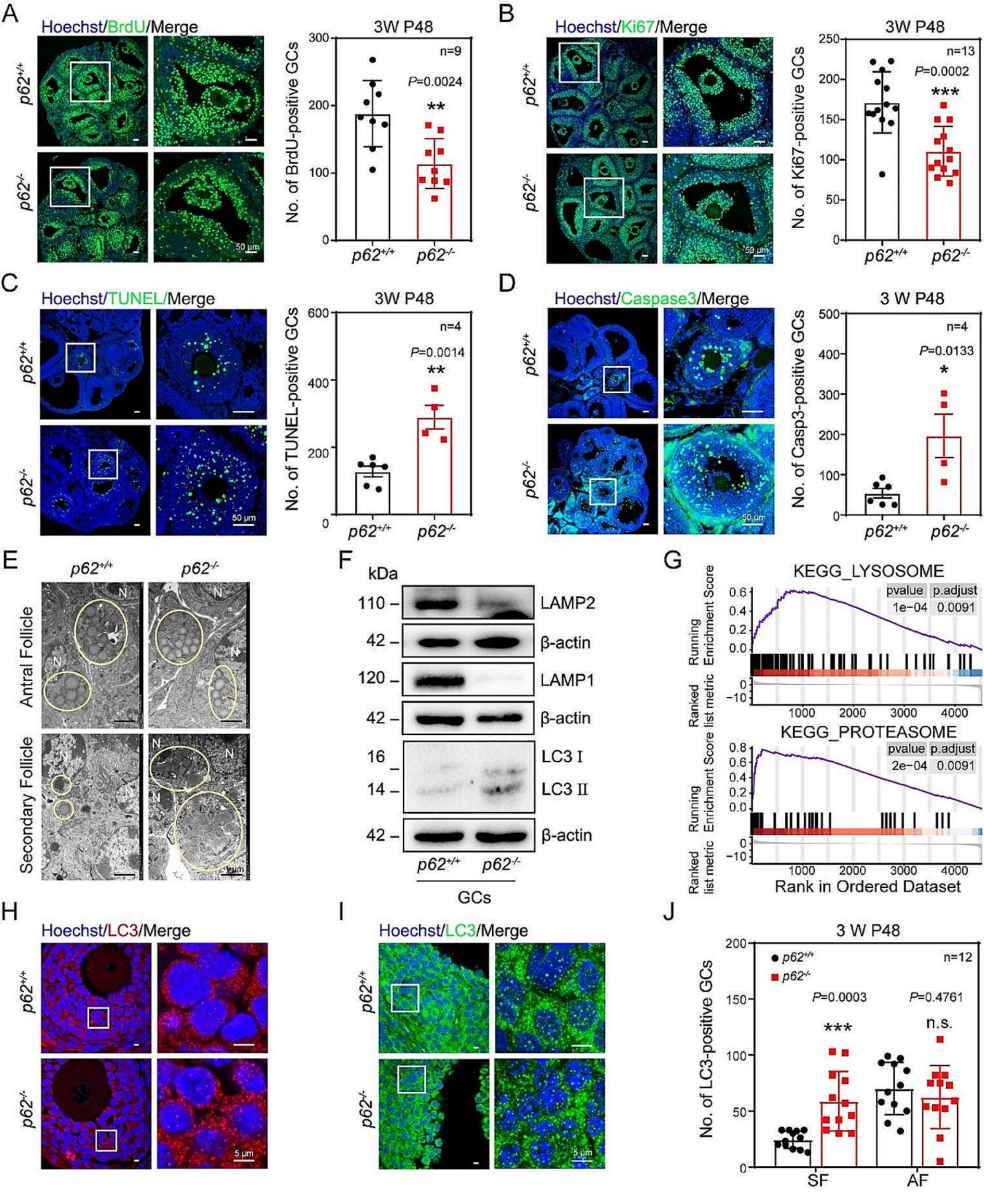
Knockout of *p62* in GCs resulted in decreased cell proliferation, increased apoptosis and blocked autophagic flux. (**A**) Cell proliferation indicated by BrdU-positive GCs in mice ovaries. Scale bar: 50 μm. BrdU: green; Hoechst: blue. Statistics data in the right (*n* = 9). (**B**) Cell proliferation indicated by Ki67-positive GCs in mice ovaries. Scale bar: 50 μm. Ki67: green; Hoechst: blue. Statistics data in the right (*n* = 13). (**C**) Cell apoptosis indicated by TUNEL signals in mice ovaries. The apoptosis signals in GCs were upregulated after *p62* deletion. Scale bar: 50 μm. TUNEL: green; Hoechst: blue. Statistics data in the right (*n* = 4). (**D**) The level of Caspase3 protein in mice ovaries. Increased level of cleaved-caspase3 in ovaries of *p62*^*−/−*^ mice indicated the upregulated apoptosis signals. Scale bar: 50 μm. Cleaved-caspase3: green; Hoechst: blue. Statistics data in the right (*n* = 4). (**E**) Autophagy vesicles observed under electron microscope. The white circle shows autophagic vesicles. N: nucleus. Scale bar: 1 μm. (**F**) Protein expression of LC3, LAMP1 and LAMP2 in GCs (*n* = 3). (**G**) GSEA analysis of lysosomal pathway and proteasomal pathway. (**H**, **I**) LC3 puncta aggregation was observed in GCs of SFs (**H**) and AFs (**I**). LC3: red/green, Hoechst: blue. Scale bar: 5 μm. (**J**) Statistics data of H and I (*n* = 12). The boxed regions in the left panels were magnified in the figure

To investigate why the GC layers were reduced after knockout of *p62*, the biomarkers of cell proliferation of GCs were assessed. Immunofluorescence staining showed that the numbers of BrdU-positive (Fig. 3A) or Ki67-positive (Fig. 3B) in *p62*^*−/−*^ mice were lower than those found in the *p62*^*+/+*^ mice. In addition, western blotting showed that the espression of PCNA, a cell proliferation marker, was downregulated in both whole ovaries (Fig. S3A) and isolated GCs (Fig. S3B) of *p62*^*−/−*^ mice. These results indicated that the proliferation of GCs in *p62*^*−/−*^ was decreased. In contrast, we found that after 48 h of PMSG induction, more GCs with TUNEL-positive signals (Fig. 3C) and cleaved Caspase-3-positive cells (Fig. 3D) were presented in *p62*^*−/−*^ mice than in control mice. The cleaved Caspase-3 protein levels in both the whole ovary (Fig. S3C) and isolated GCs (Fig. S3D) of *p62*^*−/−*^ mice increased significantly.

To further explore the reasons why the number of AFs reduced after *p62* depletion at the gene expression and protein levels, GCs of *p62*^*+/+*^ and *p62*^*−/−*^ mice primed with PMSG for 48 h were collected for mass spectrum analysis. The results showed that 340 and 154 proteins were upregulated and downregulated, respectively, in *p62*^*−/−*^ mice compared to control (Fig. S3E). Remarkably, the mass spectrometry data showed enriched of lysosomal and proteasomal signaling pathway molecules in *p62*^*−/−*^ mice (Fig. 3G). Specifically, according to the heatmap results, the protein expression of the lysosomal membrane proteins LAMP1 and LAMP2 was significantly downregulated (Fig. S3G). The overall level of proteasome-associated protein decreased (Fig. S3H). Additionally, Gene Ontology (GO) analysis showed the most significant differences in biological process (BP), cellular component (CC) and molecular function (MF) enrichment data focused on autophagy, lysosome, and ubiquitin binding (Fig. S3F). In conclusion, under the condition of specific knockout of *p62* in the GCs, the cell fate of the GCs was greatly disturbed because GC proliferation was decreased, apoptosis was increased, autophagic flux was blocked, and the proteasome pathway was abnormal.

To find more clues to support the findings, electron microscopy assays were performed to investigate whether p62 is essential for autophagy activity in the GCs during AFs formation. The results showed that the number of autophagic vesicles in the GCs of SFs of *p62*^*−/−*^ mice was significantly increased (Fig. 3E). In addition, the numbers of LC3II-positive GCs, indicated by immunofluorescence dots, increased mostly in the GCs of SFs rather than AFs (Fig. 3H-J). Furthermore, the LC3 level was elevated in GCs (Fig. 3F). Similarly, the protein levels of LAMP1 and LAMP2 were both decreased, which was consistent with the mass spectrometry data (Fig. 3F, S3G). Therefore, autophagy flow was inhibited in the ovarian GCs of *p62*^*−/−*^ mice. In addition, RNAi of *p62* (*si-p62*) was applied in FSH-stimulated KGN cells in vitro. Forty-eight hours after *si-p62* treatments, we found the same results, including proliferation levels were reduced and autophagy levels were blocked (Fig. S7A, B). Therefore, follicle GCs ceased proliferation on the one hand and initiated apoptosis and autophagy on the other after *p62* was knocked out in mouse ovarian GCs.

### The level of ubiquitination in the GCs was reduced after deletion of ***p62***

**Fig. 4:**
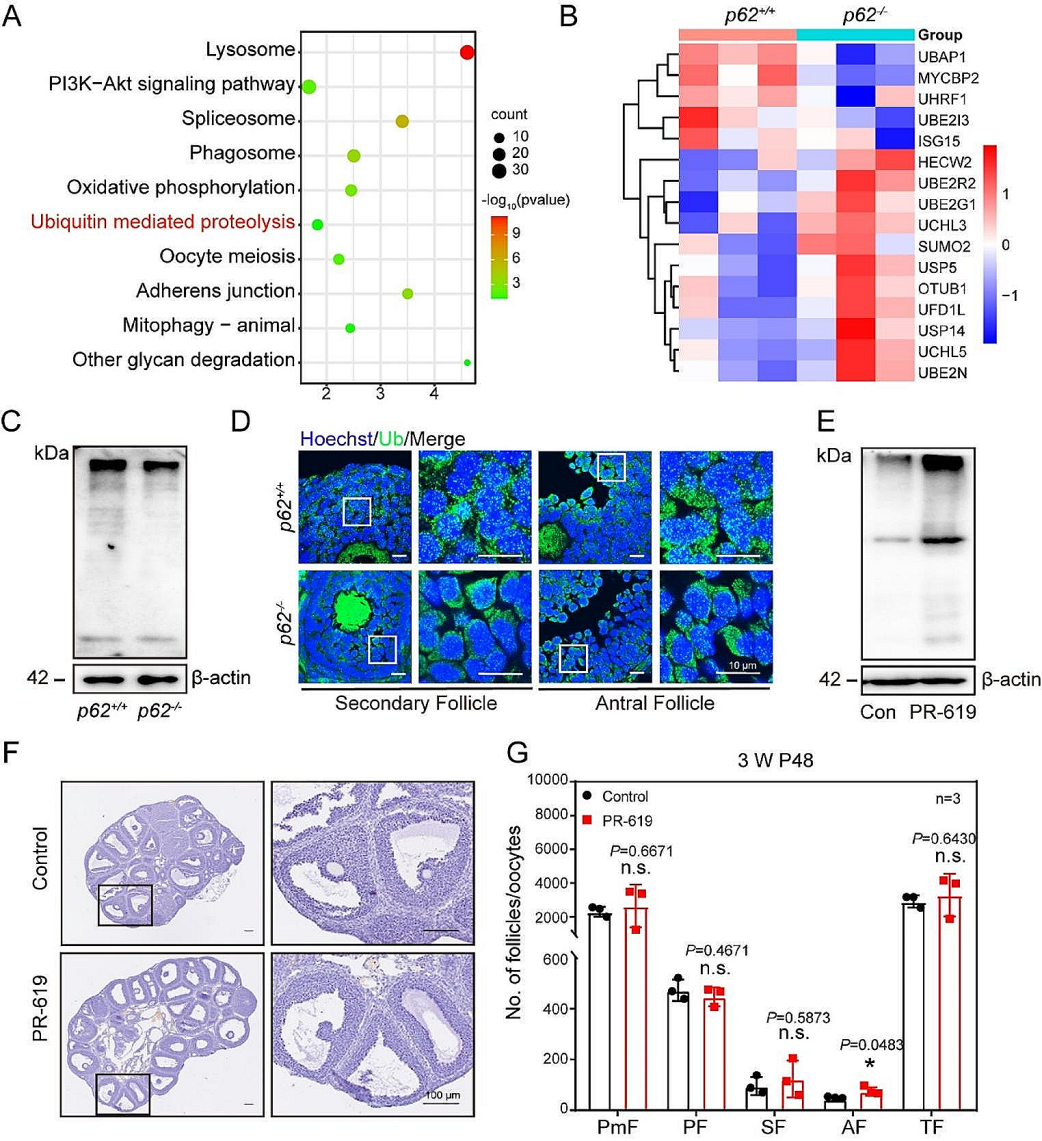
The level of ubiquitination was reduced in *p62* knockout GCs. (**A**) KEGG enrichment showed significantly altered cellular activity after *p62* deletion. (**B**) Heatmap showed the expression levels of differentially expressed genes related to ubiquitination in GCs. (**C**) Western blotting for the ubiquitination, and β-actin (internal control) levels in GCs of ovaries (*n* = 3). (**D**) Ubiquitination (Ub) in the GCs indicated by immunofluorescence in three-week-old *p62*^*+/+*^ and *p62*^*−/−*^ mice ovaries. Ub: green; Hoechst: blue. Scale bars: 10 μm. (**E**) Western blotting for Ub in KGN of PR-619 and control group. (**F**) The hematoxylin-stained ovary sections of control and PR-619 mice. The boxed region of each figure was magnified accordingly. Scale bar: 100 μm. (**G**) Statistics of the numbers of PmFs, PFs, SFs and TFs in ovaries at P0 (*n* = 3)

The above results implied that *p62* deletion affected both ALS and UPS protein degradation pathways in GCs simultaneously. The commonality of these two signaling pathways is that the degraded protein requires ubiquitination. Consequently, KEGG analysis of the mass spectrometry data showed that ubiquitin-mediated proteolysis was enriched (Fig. 4A). Similarly, the ubiquitination-related genes were enriched according to the mass spectrum data (Fig. 4B). Further, GO analysis revealed enrichment of ubiquitination-related pathways, especially K63-linked polyubiquitin modification-dependent protein binding (Fig. S4F). Compared with *p62*^*+/+*^ mice, *p62*^*−/−*^ mice showed significantly fewer ubiquitin-positive fluorescence puncta in SFs and AFs (Fig. 4D, S4E) as well as in GCs (Fig. 4C). The occurrence of the two most common forms of polyubiquitin, both K48-linked and K63-linked polyubiquitination, was decreased in *p62*^*−/−*^ mice after PMSG priming (Fig. S4A-D, S4G). Furthermore, we examined KGN cells with *si-p62*, and western blotting results showed that ubiquitination (Ub), K63-linked and K48-linked polyubiquitin levels were significantly decreased (Fig. S4H). The preliminary results suggested that the absence of *p62* in GCs leads to a decreased ubiquitination level.

Strikingly, when we injected three-week-old mouse ovaries with 75 µM PR-619, a nonspecific inhibitor of the deubiquitylation enzyme, and primed them with PMSG for 48 h, the ubiquitination level was increased compared to that in the controls (Fig. 4E). In addition, the number of AFs in the PR-619 group was significantly higher than that in the control group (Fig. 4F, G). The findings imply that the level of ubiquitination in the ovaries affects follicle development.

### The potential of cell differentiation was impaired in GCs after deletion of *p62*

**Fig. 5:**
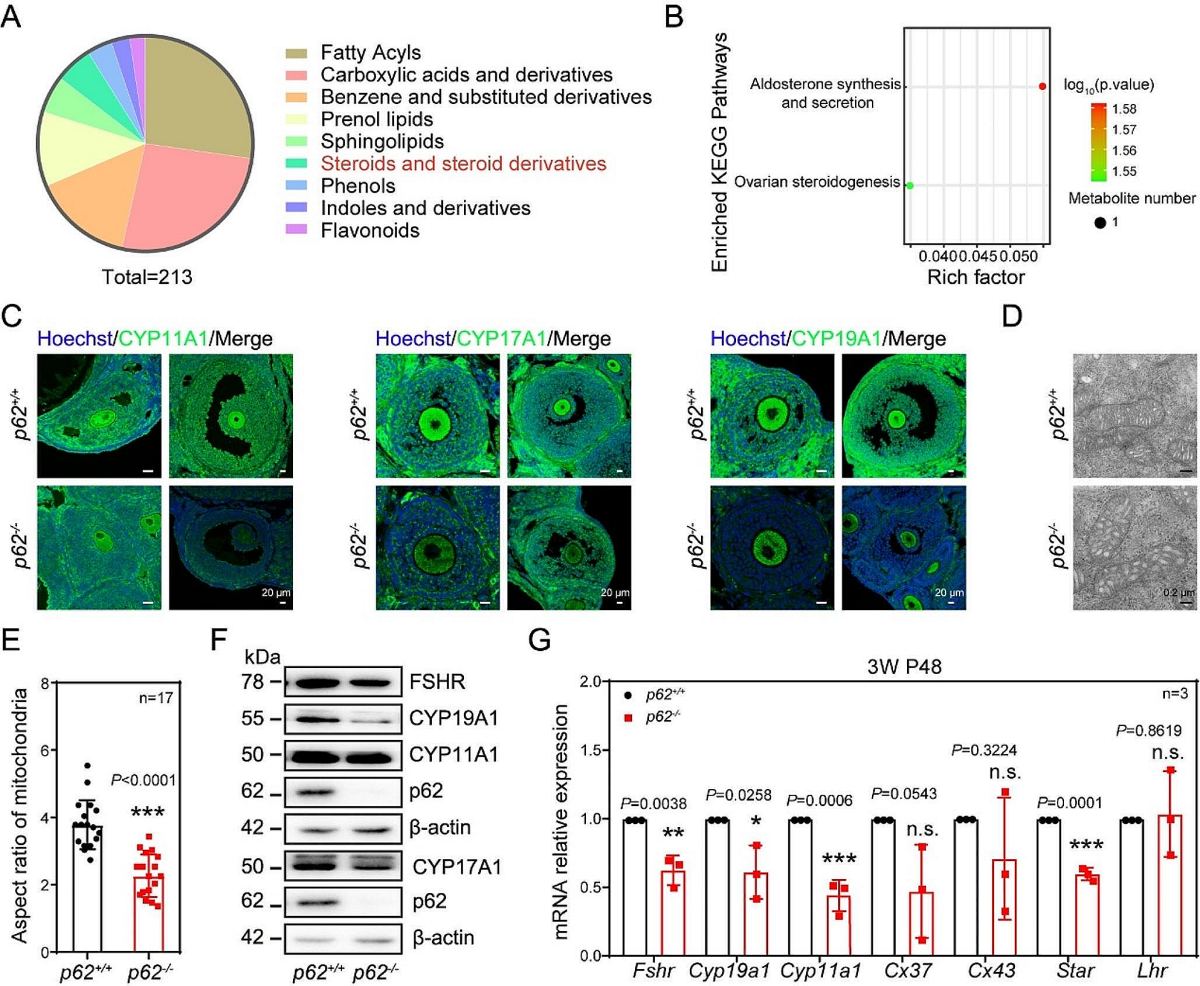
The cellular differentiation capacity was reduced in *p62* knockout GCs. (**A**) The analysis of metabolomics. (**B**) Conjoint analysis of RNA-seq and metabolome sequencing data. (**C**) The protein expression of CYP11A1, CYP17A1 and CYP19A1 in GCs of SFs and AFs was detected by immunofluorescence assays. CYP11A1、CYP17A1、CYP19A1: green; Hoechst: blue. Scale bar: 20 μm. (**D**) Electron microscope observation of mitochondria in the GCs of either *p62*^*+/+*^ or *p62*^*−/−*^ mice. Scale bar: 0.2 μm. (**E**) The aspect ratio of mitochondria (*n* = 17). (**F**) Cell differentiation-related proteins CYP11A1, CYP19A1, and FSHR in GCs. (G) Genes related to GC differentiation after *p62* deletion were examined (*n* = 3)

To investigate why AF development was arrested in *p62*^*−/−*^ mice, the ovaries of *p62*^*+/+*^ and *p62*^*−/−*^ mice treated with PMSG for 48 h were used for RNA-seq. The results showed that 33 genes were upregulated and 13 genes downregulated, respectively (Fig. S5A). In addition, we noticed that loss of *p62* in GCs caused abnormal expression of cellular metabolic-related genes (Fig. S5B). Specifically, the lipid and lipid-like metabolism-related genes accounted for the largest proportion of altered expression (Fig. S5C). A detailed analysis of this category focused on the fatty acids, carboxylic acids and their derivatives as well as steroids and steroid derivatives (Fig. 5A). These data suggested that specific deletion of *p62* in GCs caused metabolic homeostasis disorder in the ovaries. Furthermore, to determine which kind of substance metabolism was abnormal, a joint analysis of RNA-seq sequencing data and the metabolic data were carried out. The results showed that the most significant differences focused on steroid signaling pathways (Fig. 5B). Collectively, these results implied that the steroid metabolic process that correlated to sex hormone production in the GCs was impaired after the deletion of *p62* in GCs.

To identify whether specific depletion of *p62* resulted in cellular differentiation failure of GCs, some of the molecules that are pivotal for supporting GC functions were evaluated. The examined markers included representative markers of either the steroidogenesis or the FSH responsiveness, such as FSH receptor. In terms of steroid generation, we found that the protein levels of CYP11A1, CYP17A1 and CYP19A1 in *p62*^*−/−*^ mice were simultaneously lower than those found in *p62*^*+/+*^ mice (Fig. 5C and F, S5D-F). Furthermore, the mRNA expression of *Fshr*, *Cyp19a1*, *Cyp11a1*, and *Star* was also significantly downregulated in *p62*^*−/−*^ mice (Fig. 5G). Notably, we found that the crista swelling occurred in the mitochondria of GCs according to our electronic microscope results (Fig. 5D, E), which implied that metabolism in these cells may have been impaired after *p62* deletion. Additionally, we found that the expression of some GC differentiation-related proteins was downregulated in the *si-p62* group, including CYP11A1, CYP17A1, CYP19A1, and FSHR, in accordance with the results found in *p62* knockout mice (Fig. S7A-C). Therefore, after *p62* knockout, the differentiation ability of follicular GCs decreased.

### The level of WT1 was greatly upregulated after deletion of p62

**Fig. 6:**
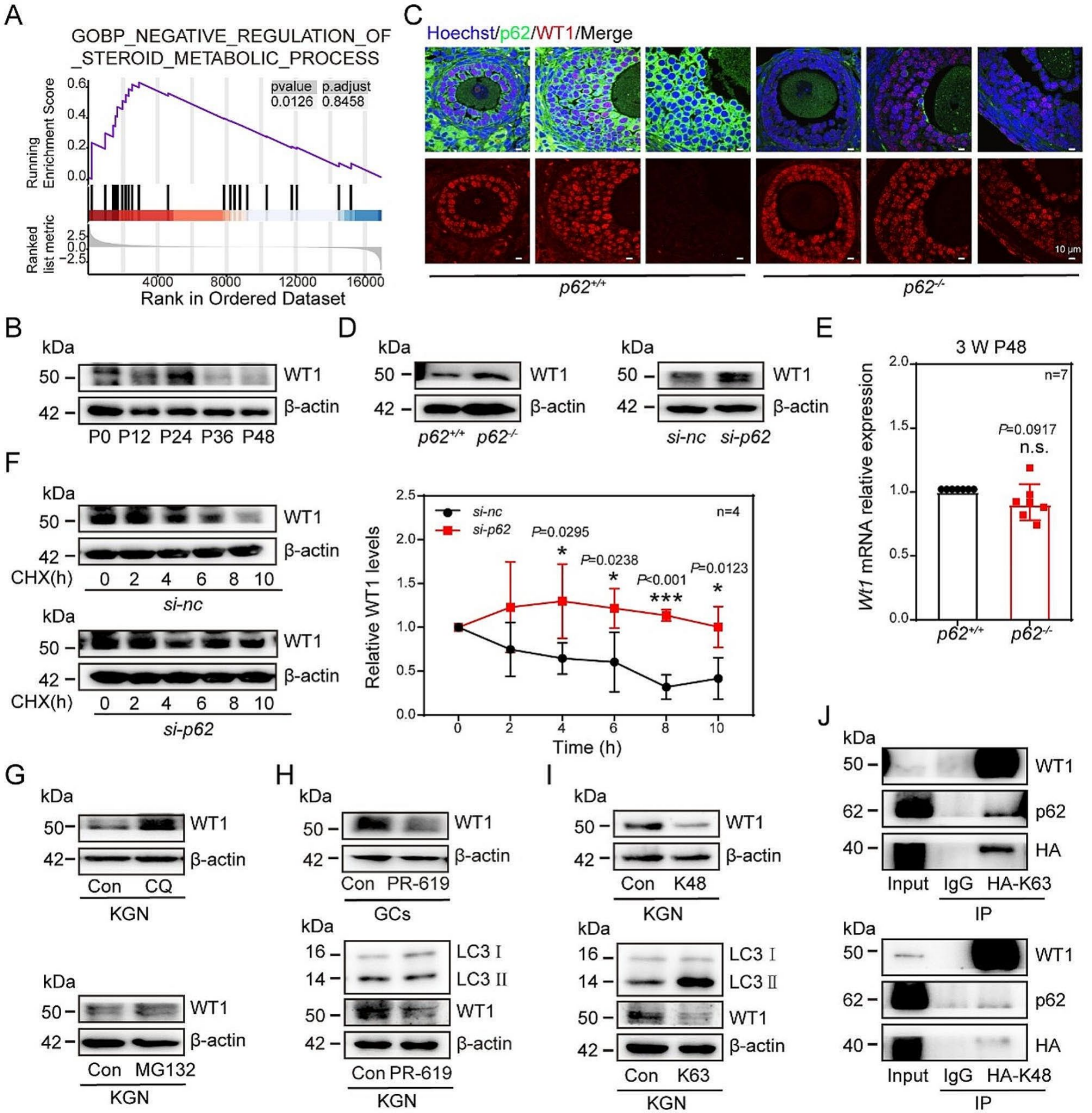
WT1 was greatly upregulated after deletion of *p62*. (**A**) Negative regulation of steroid metabolic process pathways by GO_BP analysis. (**B**) The expression pattern of WT1 protein in the isolated GCs was determined after PMSG induction (*n* = 3). (**C**) The expression levels of WT1 in GCs of SFs and AFs examined by immunofluorescence. WT1: red; p62: green; Hoechst: blue. Scale bar: 20 μm. (**D**) The expression pattern of WT1 protein was upregulated in *p62* knockout GCs and *p62* knockdown KGN cells (*n* = 3). (**E**) The mRNA of *Wt1* in GCs of *p62*^*+/+*^ and *p62*^*−/−*^ mice (*n* = 7). (**F**) Knockdown of *p62* blocked the degradation of WT1. CHX, cycloheximide, 10 µg/mL. (**G**) The protein levels of WT1 in KGN cells after CQ or MG132 treatments, respectively (*n* = 3). (**H**) Western blotting for WT1 in KGN and GCs of control and PR-619 group (*n* = 3). (**I**) The protein levels of WT1 in KGN cells after PCMV-HA-hub K48R or PCMV-HA-hub K63R treatments, respectively (*n* = 3). (**J**) The co-IP results after KGN cells were treated with PCMV-HA-hub K48R or PCMV-HA-hub K63R plasmid

Consistent with these findings, the negative regulation items of the steroid metabolic process were enriched according to our RNA-seq analysis (Fig. 6A). Given that the pivotal role of WT1 in GCs was to regulate GC differentiation, we further investigated whether the level of WT1 in GCs during secondary-antral follicle transformation was changed in *p62*^*−/−*^ mice. The results showed that in the control mice, WT1 increased gradually from 0 h to 12 h through 24 h after PMSG treatment but tended to decrease after 36 h to 48 h (Fig. 6B). The level of WT1 in ovarian of *p62*^*−/−*^ mice, however, did not change significantly in SFs. Instead, WT1 expressed at a higher level in AFs as compared to the *p62*^*+/+*^ mice (Fig. 6B, S5G). Furthermore, the overall expression level of WT1 protein (Fig. 6D, S5H), rather than *Wt1* mRNAs, in ovarian GCs of *p62*^*−/−*^ mice was increased (Fig. 6E). And, in KGN cell, *p62* knockdown also caused the protein expression level of WT1 to increase (Fig. 6D). After transfection with *si-Wt1*, the expression of CYP11A1, CYP17A1, CYP19A1 and STAR was upregulated, indicating that WT1 could indeed affect GC differentiation, which was consistent with previous conclusions (Fig. S7D). Therefore, it is speculated that p62 may affect the protein expression level of WT1 in GCs under physiological conditions. To clarify whether p62 degrades the WT1 protein in a posttranscriptional manner instead of at the transcriptional level, KGN cells were treated with the protein synthesis inhibitor cycloheximide (CHX) and either *si-nc* or *si-p62*. The results showed that the reduction in WT1 protein expression in *si-p62*-treated KGN cells was obviously delayed (Fig. 6F), suggesting that *si-p62* treatment enhanced the stability of the WT1 protein. Based on this, it is concluded that p62 affects GC differentiation by affecting the degradation of WT1.

Furthermore, to confirm whether p62 affects WT1 expression via ubiquitination, we cultured KGN cells with either autophagy inhibitor CQ or the proteasome inhibitor MG132 in vitro. The results showed that the protein level of WT1 increased in both groups (Fig. 6G). These results suggested that both autophagy and proteasome pathways influence the expression of WT1. Specifically, the WT1 protein levels decreased in KGN and GCs after PR-619 treatment (Fig. 6H).

To further clarify how p62 works by modulating ubiquitination in GCs, K48-linked ubiquitination, which is mostly involved in the proteasome degradation pathway, and K63-linked ubiquitination, which promotes autophagy, were studied. Then, the level of WT1 decreased when either PCMV-HA-hub K48R or PCMV-HA-hub K63R was transfected into KGN cells (Fig. 6I). Coimmunoprecipitation (co-IP) assays showed that p62 could bind to ubiquitination-modified WT1 in both the K48 and K63 groups (Fig. 6J). In summary, p62 affects the degradation of WT1 by affecting the ubiquitination of both K48-linked and K63-linked proteins in GCs.

### USP5 regulated the level of WT1 in the GCs of *p62* knockout mice

**Fig. 7:**
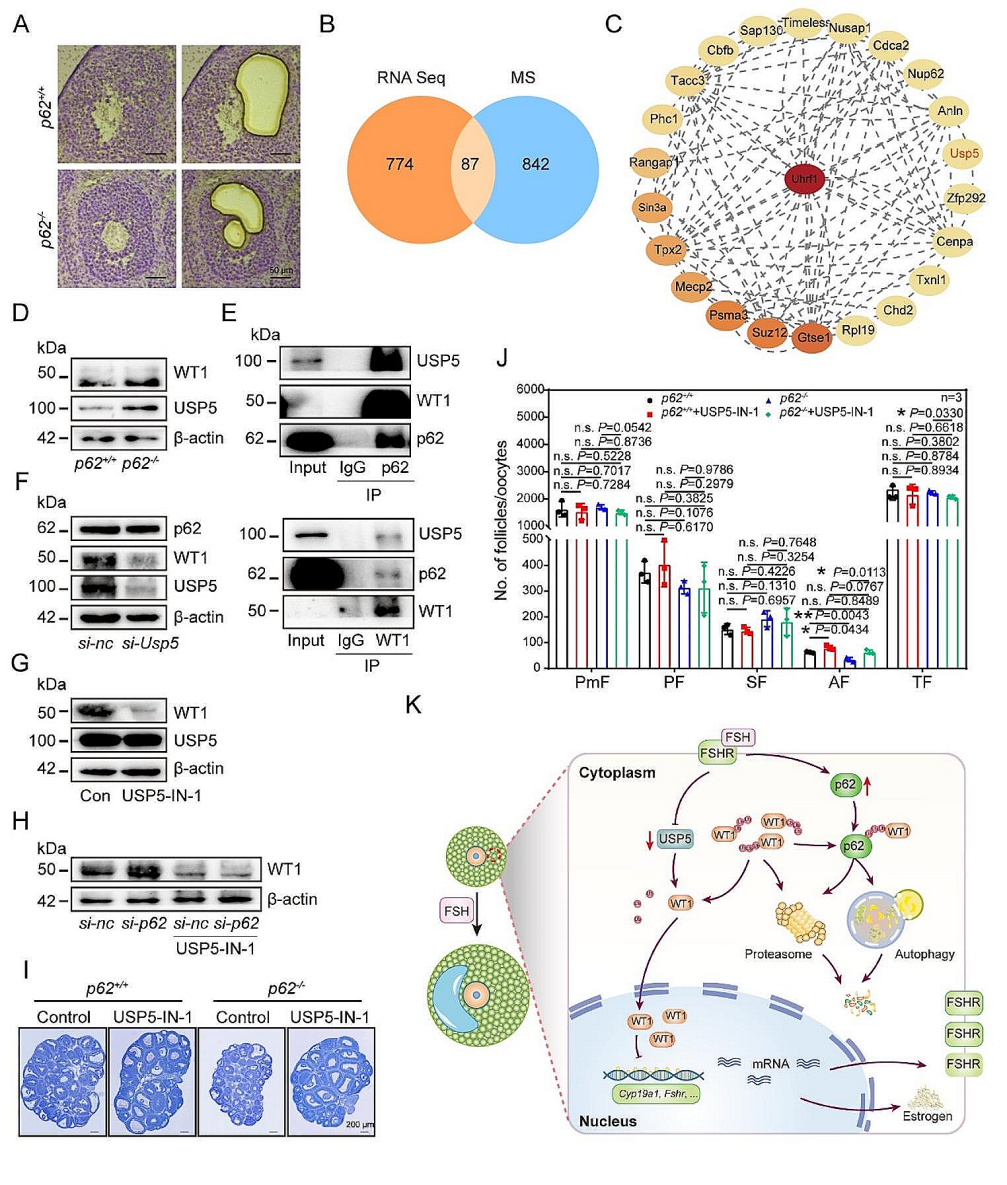
Available USP5 upregulated the expression of WT1 in the GCs of *p62* knockout mice. (**A**) A schematic of the laser capture microdissection procedure of GCs. After removing the oocyte, the GCs of each SF were cut and collected for analysis. Scale bars: 50 μm. (**B**) Overlap between mass spectrometry genes and transcriptome sequencing genes. (**C**) PPI analysis was performed on 87 coincident genes in B. The top 23 genes were selected for mapping. (**D**) Protein levels of WT1 and USP5 in GCs of *p62*^*+/+*^ and *p62*^*−/−*^ mice. (**E**) The protein interactions between p62 and WT1 or USP5 were demonstrated. (**F** and **G**) Protein level of USP5 and WT1 after KGN cells were treated by *si-Usp5* (**F**) or USP5-IN-1 (**G**), respectively (*n* = 3). (**H**) Protein level of USP5 and WT1 after *si-p62* plus simultaneous USP5-IN-1 and FSH treatments for 48 h (*n* = 3). (**I**) Statistics data of the number of follicles in ovaries after treatments (*n* = 3). (**J**) Representative structure of ovaries after USP5-IN-1 treatments in vivo. Scale bar: 200 μm. (**K**) Proposed model for the role of p62 in regulating ovarian GC differentiation

To identify the major regulators of ubiquitination in GCs correlated with *p62* deletion, we then performed spatial transcriptome sequencing using SFs collected from either *p62*^*+/+*^ or *p62*^*−/−*^ mice. As shown in Fig. 7A, we used microdissection techniques to harvest GCs from the individual SFs of three-week-old mice at the P48 time point and then used them for RNA-seq (Fig. S6A). Then, the sequencing data were analyzed by GO and KEGG. The results showed that the proteasome and steroid synthesis signaling pathways were both enriched (Fig. S6B-D). Specifically, by comparing the differential genes derived from this database with our mass spectrum data, 87 overlapping genes were identified (Fig. 7B).

Furthermore, the protein-protein interaction (PPI) analysis of these overlapping genes was conducted to identify highly correlated molecules (Fig. 7C). USP5 not only regulated the ubiquitination level but also affected the expression of WT1. Additionally, Heatmap analysis and western blotting confirmed that after the deletion of *p62*, the level of USP5 protein in the *p62*^*−/−*^ GCs was upregulated (Fig. 7D, S6E). Moreover, the USP5 protein localized mainly in the cytoplasm of GCs (Fig. S7G). The expression level of USP5 decreased gradually in response to FSH treatment (Fig. S7F). Notably, the results of the IP experiment and protein-protein docking experiment showed that there was an interaction among p62, USP5 and WT1 (Fig. 7E, S7I).

To further confirm the correlation between the p62, USP5 and WT1, USP5-IN-1, a potent ubiquities USP5 inhibitor, was used KGN cells in culture medium. On the one hand, in cultured KGN cells, silencing of *Usp5* and inhibiting USP5 with USP5-IN-1 simultaneously resulted in decreased WT1 expression but increased pan-ubiquitination levels (Fig. 7F and G, S7H). On the other hand, applying USP5-IN-1 to the cultured KGN cells reversed the changes caused by *si-p62* treatment (Fig. 7H). After transfection with *si-Usp5*, the expression of CYP17A1, CYP19A1 and STAR were upregulated, indicating that USP5 could indeed affect GC differentiation (Fig. S7E). These results suggested that the blockage of GC differentiation caused by *p62* deficiency may be reversed, at least in part, by restricting the USP5 level.

Then, 70 µM USP5-IN-1, was injected into the 3-week-old mice that were primed with PMSG simultaneously. The WT1 protein level decreased (Fig. 7G) and the ubiquitination level increased (Fig. S7H) in KGN after USP5-IN-1 treatment. The results showed that only the number of AFs in the USP5-IN-1 group was significantly higher than that in the control group at P48 (Fig. 7J). To clarify whether inhibition of USP5 in the *p62*^*−/−*^ mice had a similar effect on follicle development, we injected USP5-IN-1 into *p62*^*+/+*^ and *p62*^*−/−*^ mice. The results showed that inhibition of USP5 effectively reversed the phenotype of AF reduction caused by *p62* deletion (Fig. 7I, J).

In conclusion, the action of p62 in the GCs was mediated indirectly through USP5, which finally affected the general ubiquitination level of WT1 via degradation of the protein through either the UPS or ALS. Specifically, the homeostasis of ubiquitinated WT1 and degradation relies on binding of WT1 to p62 in GCs. Once p62 level is reduced, the highly expressed USP5 deubiquitinated WT1 to prevent WT1 degradation. Consequently, the accumulation of WT1 in GCs inhibits cell differentiation as well as preantral-to-antral follicle transformation by restricting the expression of multiple differentiation-related genes (Fig. 7K).

## Discussion

In this study, p62 showed a downward trend in GCs of POR patients, suggested that p62 may be a potential factor in the diagnosis of POR patients. Studies in mice have found that the burst of p62 in FSH-induced GCs is important for supporting AF formation during the fast growth of SFs. Genetic deletion of *p62* in mouse GCs resulted in impaired fertility, cell cycle disruption and impaired cell differentiation by maintaining an increased level of ubiquitination of cell differentiation-related proteins such as WT1. Consequently, it is concluded that under physiological conditions, FSH increases the activity of p62 to counter the role of USP5 in controlling the ubiquitination of WT1 in GCs and thus promotes the degradation of WT1 through either the UPS or ALS. The findings imply that FSH-induced general GC differentiation-related gene expression depends on, at least in part, a decrease in the level of WT1 controlled by the p62-USP5 couple, which is consistent with previous reports explaining that WT1 regulates granulosa cell differentiation [38–42].

POR accounts for 9-24% of all infertile women, and the probability increases gradually with the increase of childbearing age, which seriously threatens the health and life of patients [1, 2]. However, most pathogenic variants in POR patients are uncertain. In this study, we selected patients with POR for evaluation who had significantly reduced mRNA levels of p62 in GCs. Subsequently, we verified the phenotype of *p62* knockout mice was consistent with that of POR patients, including decreased estrogen levels, decreased ovulation, and decreased fertility. These results remind us that reduced p62 expression levels may be a potential causative agent in patients with POR. Based on our data, the causes of AF reduction and low fertility in *p62*^*−/−*^ mice appear to be complex. Studies including ours have proven that, as WT1 is an important zinc finger nuclear transcription factor, the degradation of WT1 is important for promoting GC differentiation and responding to FSH. According to reports, WT1 is important for early follicle growth since mutation of *Wt1* in mice results in subfertility, impaired early follicle defects and aberrant expression GC differentiation-related genes ^33, 35^. The accumulation of WT1 in GCs inhibits the expression of differentiation-related genes, such as FSHR and CYP19A1, resulting in defective differentiation of GCs [23, 39–42]. It has been reported that deficiencies in the expression of steroidogenic enzymes, aromatase, and FSHR have been reported in POR patients, which is consistent with the results in *p62*^*−/−*^ mice [54]. It is speculated that there is a relationship between the accumulation of WT1 and POR patients. Consistent with this, we confirmed that the downregulation of WT1 in GCs is pivotal for FSH-induced follicle growth and dominant follicle selection. The decrease in WT1 could be complex. For instance, we have proven that in response to FSH, the decrease in the level of WT1 in GCs depends not only on the downregulation of *Wt1* transcription by LSD1 but also on the degradation of WT1 protein via autophagy [64]. As a complement to previous discoveries, this study further confirms that ubiquitinated WT1 is degraded by the proteasomal degradation mechanism during follicular development. Altogether, the findings of the present study confirm that WT1 plays a pivotal role in controlling the process of GC differentiation in SFs in response to FSH.

This study demonstrated that the p62-USP5 couple acts as an important downstream effector of FSH in directing the differentiation of GCs by modulating the ubiquitination level of WT1. Importantly, as an intermediate protein, p62 is involved in the clearance of ubiquitination substrates, which is particularly important for the maintenance of protein homeostasis [55–58]. In *C. elegans* and *Drosophila*, either p62 or its homolog is needed for the aggregation of cytoplasmic ubiquitinated proteins and for autophagic clearance [59, 60]. Consistently, p62 reduces citrate levels in porcine cumulus cells via Lys63-linked polyubiquitin of ATP citrate lyase and affects the quality of oocyte maturation [53]. In agreement, this study showed that p62 was localized in the GC cytoplasm during FSH induction and was important for the degradation of proteins. Consequently, prevention of deubiquitylation with PR-619 not only proved that the ubiquitin levels in the ovaries affect GC differentiation but also that *p62* knockout reduced the ubiquitination level of GCs, which resulted in abnormalities in both the UPS and ALP protein degradation pathways. Therefore, it is assumed that the stabilization of ubiquitination levels mediated by p62-USP5 in GCs during follicular dominant selection is necessary to clear unneeded proteins induced by FSH induction, thereby regulating normal follicular development.

Interestingly, p62 is deeply involved in cyclic follicle development through the hypothalamus–pituitary–ovary axis. The specificity of p62 in female reproduction is that it not only participates in regulating LH secretion in the pituitary [52, 61, 62], but also acts in response to FSH induction in ovarian GCs, as shown in this study. For instance, systematic knockout of *p62* in mice resulted in obesity accompanied by female infertility, including pituitary dysfunction, as indicated by decreased LH levels during estrus, and ovarian dysfunction, as indicated by abnormal menstrual cycles, follicular formation, anovulation, and steroidogenesis [62, 63]. At the molecular level, p62 in the pituitary regulates LH secretion through the gonadotropin-releasing hormone-p62-mitochondrial oxidative phosphorylation-Ca^2+^/ATP-LH signaling pathway [62]. Our work also confirmed that during PMSG induction, the levels of p62 in the GCs of both SFs and AFs changed dynamically in vivo. However, when p62 was deleted in GCs, the formation of AFs was significantly impaired, and estrogen secretion as well as LH levels in the sera were all reduced, which ultimately led to decreased fertility in females. These findings imply that p62, as a multifunctional protein in somatic cells, is indispensable for female reproduction. Although USP5 and p62 are both FSH responsive and share WT1 as the same target, we are still uncertain about the relationship between the two proteins. Therefore, more studies are needed to clarify the relationship between p62 and USP5 in GCs.

In summary, p62 plays an essential role in female reproduction by participating in FSH-induced AF formation. A follicle growth-dependent increase in the ubiquitination level of WT1 modulated by the p62-USP5 couple ensures FSH-induced GC differentiation by promoting the expression of multiple genes during AF formation, which is pivotal for dominant follicle selection. The study also emphasizes the importance of coordination between the protein post-translational modification conveyed by ubiquitination of the key cellular differentiation molecules and gonadotropin stimulation in determining the fate of growing follicles. p62 was supposed to be the candidates of protein post-translational modification in POR diagnosis and pathology study.

## Materials and methods

### Animals

C57BL/6 mice were purchased from the National Institute of Biological Sciences (Beijing, China). We hybridized Foxl2-Cre mice by crossed with *p62*^*flox/flox*^ mice to obtain *p62*^*flox/flox*^; *Foxl2-Cre* mice [64]. Thus, the resulting *p62*^*flox/flox*^; *Foxl2-Cre* mice were referred to as *p62*^*−/−*^ mice and *p62*^*flox/flox*^ females were used as control mice, namely *p62*^*+/+*^ mice. The *Foxl2-Cre* mice were a gift from Professor Fei Gao, Institute of Zoology, Chinese Academy of Sciences (Beijing, China). Primers to identify knockout mouse genotypes are as follows: *p62-seq*-F: TGAGAAGGCAGATGGGACAGGGA, *p62-seq*-R: GCCCAGACATAAGCCACCCACCT; *foxl2-Cre*-F: TGCTTCTGTCCGTTTGC, *foxl2-Cre*-F: CCACCGTCAGTACGTGAG. All these mice were housed under controlled temperature (22 °C) and light conditions (14 h light, 10 h darkness; lights on at 07:00 a.m.) and allowed free access to chow and water. The breeding test was performed by crossing *p62*^*+/+*^ and p62^−/−^ female mice with proven fertile males for six months. All drugs were injected intraperitoneally, and the concentrations are shown as follows. PR-619 (13,814; 0.12 µg/g [body weight]), USP5-IN-1 (139,979; 0.255 µg/g [body weight]) were both purchased from MedChemExpress [65].

All procedures were conducted in accordance with the guidelines of and approved by the Animal Research Committee of the China Agricultural University.

### Fertility test

Five six-week-old *p62*^*−/−*^ and the *p62*^*+/+*^ female mice were continuously mated with eight-week-old fertile males over 25 weeks. The numbers of pups and litters were recorded, and fertility rates were calculated respectively [64].

### Estrus cycle detection

The estrous cycle is divided into different stages, including diestrum, proestrus, estrus, and metestrus. These stages can easily be determined by examining washes or cell smears of the vagina [66]. A small amount of saline was aspirated with an eyedropper into the vaginal opening of the five-week-old *p62*^*−/−*^ and the *p62*^*+/+*^ mice, and a drop was gently aspirated back for a smear. After drying, the smear was fixed with 95% ethanol and observed under the microscope after hematoxylin-eosin staining. Mice vaginas were detected every morning and monitored continuously for three weeks.

### Superovulation

To calculate the number of ovulations in mice, the three-week-old *p62*^*−/−*^ and the *p62*^*+/+*^ mice were subjected to ovulation induction: intraperitoneally injected with 5 IU PMSG (Sansheng Pharmaceutical Co., Ltd., 110,914,564), followed by 46 h later by 5 IU hCG (Ningbo Sansheng Pharmaceutical Co., Ltd., 110,911,282). After 14 h, the fallopian tubes were removed and the ampulla was cut to obtain oocyte/cumulus masses. The oocyte-cumulus masses were dispersed with 0.3% hyaluronidase (Sigma-Aldrich, 37326-33-3) and the cumulus cells were shed. The number of oocytes was counted [67].

### Ovary isolation and GCs collection

The mice were killed by cervical dislocation. The ovaries were obtained from the ovarian sacs of the dorsal kidney, placed in phosphate buffered saline (PBS) (10 mM, pH 7.4), and the fat around the ovaries was stripped clean under a stereomicroscope (COIC, China, ZSA302). After that, the ovaries were placed in clean PBS and the follicles were punctured with a needle under a stereomicroscope to release GCs. Oocytes are generally separated by the mouth-controlled glass pipette, and the remaining liquid was centrifuged to obtain GCs. The process of Ovary isolation and GCs collection was described in our previous study [68].

### Hormone level tests

The levels of FSH, LH and E2 in the blood are very important in the detection of ovarian function. Blood was taken from the eyeballs of mice under anesthesia. The sera of each mouse were centrifuged (1000 x g, 5 min, 4℃). Serum hormone levels were measured by Beijing North Institute of Biotechnology Co., Ltd. (Beijing, China) [69].

### Western blotting analysis

For protein expression analysis, we isolated and collected the ovaries. For adherent cells, we digested the samples with 0.25% trypsin and collected the samples. The ovaries and cells were lysed by Radioimmunoprecipitation assay buffer (RIPA, 50 mM Tris–HCl [pH 7.5], 150 mM NaCl, 1% NP-40, 0.1% SDS, 1% sodium deoxycholate, 5 mM EDTA), and phenylmethanesulfonyl fluoride (PMSF, Amresco, 0754, USA) was added to the lysate. The polyvinylidene fluoride membranes (PVDF) membrane (Millipore, IPVH00010, USA) was visualized using the SuperSignal West Pico Chemiluminescence Detection System (Thermo, Prod 34,080, USA) [70].

### Antibodies

Antibodies in this study: p62 (ab56416), KI67 (ab15580), BrdU (ab6326), DDX4 (ab13840), WT1 (ab89901), Lamp2 (ab65231), CYP17a1 (ab125022), Ubiquitin (linkage-specific K48) (ab140601), Ubiquitin (linkage-specific K63) (ab179434), were all purchased from Abcam (Cambridge, UK). Antibodies in this study: LC3B (2775), Caspase-3 (9662), Lamp1 (99,437), CYP11a1 (14,217), Star (8449), Ubiquitin (58,395), were all purchased from Cell Signaling Technology (Boston, USA). Antibodies in this study: Active caspase-3 (AC033), CYP19a1 (AF6231), PCNA (AF1363), were all purchased from Beyotime Biotechnology (Shanghai, China). Other antibodies including Foxl2 (Novus Biologicals, NB100-1277), β-actin (ABclonal, AC026), FSHR (Proteintech, 22665-1-AP), 3β-HSD (Santa Cruz Biotechnology, SC-30,820), USP5 (Proteintech, 10473-1-AP), HA (Abmart, M20021). Goat anti-rabbit FITC (ZF-0311), goat anti-mouse FITC (ZF-0312), and goat anti-mouse TRITC (ZF-0313)-conjugated secondary antibodies were purchased from Zhong Shan Jin Qiao (Beijing, China).

### Nucleoplasm separation

The GCs were lysed by RIPA, and PMSF was added to the lysate. After 15 min, nucleoplasm separation was detected using a nuclear protein and cytoplasmic protein extraction kit (Beyotime Biotechnology Co., Ltd, P0027, China) according to the manufacturer’s instructions. This technique has been reported [71].

### Histological sections and follicle counts

The PmFs were defined as oocytes surrounded by monolayer squamous GCs. The PF is composed of an oocyte surrounded by a single cubic GC layer. The SF consists of an oocyte surrounded by two or more layers of cubic GCs with no visible follicular cavity. The AF contains five or more layers of GCs and has a well-defined single follicular cavity. Atretic follicles are divided into three types: GC nuclei pyknotic, follicular fluid containing cell debris; Oocyte shrinkage; the follicles collapsed and only a few GCs remained [11].

Ovarian sections were stained with hematoxylin and slides were scanned with Leica Aperio VESA8 microscope. PmFs and PFs were counted in each section. For SFs, Afs and atretic follicles only the follicles with clear oocyte nuclei were counted to exclude the effect of repeated counting. The sum of the numbers of PmFs, PFs, SFs and AFs were the total number of follicles at different developmental stages.

### Culture and transfection of KGN cell lines

Human cell line KGN was purchased from Shanghai BinSui Biological Technology Co., Ltd. KGN was cultured in DMEM basic medium (Gibco, C11995500BT) supplemented with 10% fetal bovine serum (FBS, Sigma-aldrich, F8687), 1% penicillin–streptomycin solution (Gibco, 15140-122), and stored at 37℃ in 5% CO_2_.

According to the manufacturer’s instructions, small interfering RNA (*si-p62*: F: GUCUCCGAUAUCUGUUAAUTT, R: AUUAACAGAUAUCGGAGACTT; *si-Usp5*: F: GGAGUUCUUCCUUCACCUUTT, R: AAGGUGAAGGAAGAACUCCTT; *si-Wt1*: F: AAAUUGUCACUGCUGUGUAGGTT, R: CCUACACAGCAGUGACAAUUUTT) was transfected into KGN cells using Lipofectamine 3000 Transfection Kit (Invitrogen, CN2481208) [72]. All siRNA and negative control siRNA were purchased from GenaPharma (Suzhou, China).

Unless otherwise specified, FSH (National Hormone and Peptide Program, USA; 10 µg/µL) was added to cell culture for 48 h, and other specific drug concentrations were shown as follows. CQ (17,589 A; 10 µM), MG132 (13,259; 10 µM), PR-619 (13,814; 10 µM), USP5-IN-1 (139,979; 7.5 µM) and CHX (12,320; 10 µM) were both purchased from MedChemExpress.

### RNA isolation & real-time PCR (RT-PCR)

RNAs from all ovaries and cells were extracted with TRIZOL reagent (Invitrogen, Life Technologies, USA). Then, cDNA was synthesized according to the manufacturer’s specification (Promega Reverse Transcription System, Promega, USA). RT-PCR was performed with Power SYBR Green PCR Master Mix (Roch, 61,396,500). Applied Biosystems 7300 Real-Time PCR System (Roch, USA) was used for analysis [73]. Primers are as follows: *β-actin*, F: GTGACGTTGACATCCGTAAAGA, R: GCCGGACTCATCGTACTCC; *Fshr*, F: CCTTGCTCCTGGTCTCCTTG, R: CTCGGTCACCTTGCT ATCTTG; *Lhr*, F: GACAACCTCCTCAATCTGTCTG, R: AAAGCGTTCCCTGGTATGGTG; *Cyp19a1*, F: CAAGTCCTCAAGCATGTTCCA, R: AAGGCTCGGGTTGTTGTTAAATA; *Cyp11a1*, F: AGGTCCTTCAATGAGATCCCTT, R: TCCCTGTAAATGGGGCCATAC; *p62*, F: TGTGGAACATGG AGGGAAGAG, R: TGTGCCTGTGCTGGAACTTTC; *Usp5*, F: ATGCAGTTGCCTGTACCCATGG, R: CAGTGGCACTTTCTCCTCTTCAG; *Cx37*, F: AACGGTGCTCTTCATCTTCCGC, R: GGTCATA GCAGACGTTGGTGCA; *Cx43*, F: GGTGATGAACAGTCTGCCTTTCG, R: GTGAGCCAAGTAC AGGAGTGTG; *Star*, F: GTGCTTCATCCACTGGCTGGAA, R: GTCTGCGATAGGACCTGGTTGA.

### Immunofluorescence and immunohistochemistry

The process of immunofluorescence and immunohistochemistry was described in our previous study [68]. The ovaries were fixed in 4% paraformaldehyde (PFA) overnight, embedded in paraffin wax and continuously sliced at seven microns. It was then dewaxed and rehydrated for immunofluorescence detection. Heated in 0.01% sodium citrate buffer (pH 6.0) on high (95–98 °C) for 4 min and on low for 4 min. Repeat three times and let cool to room temperature. Washed the samples three times with PBS. After 10% donkey sera (Jackson ImmunoResearch, 017-000-121) were enclosed for 1 h, the samples were incubated overnight at 4℃ with primary antibody configured with PBS. The samples were then incubated with Alexa Fluor 488- or 555-conjugated donkey secondary antibodies against mouse (Jackson, USA) and Hoechst 33,342 (Sigma, USA), at 37 °C for 1 h. Slices were sealed with an anti-fade fluorescence mounting medium (Ruitaibio, China).

For immunohistochemistry analysis, ovary sections were handled by using a rabbit streptavidin-biotin method detection system (ZSGB-Bio, SP-9001) according to the manufacturer’s protocol.

### BrdU and TUNEL assays

Two hours before sample collection, mice were injected with BrdU (Sigma-Aldrich, 11,170,376,001; 100 µg/g), with a concentration of 10 mg/kg. The ovaries were sliced and rehydrated, repaired and sealed. Then the samples were incubated with BrdU antibody (Abcam, ab1893) overnight, incubated with goat-derived secondary antibody at room temperature for one hour, washed three times with PBS, and photographed under a microscope after sealing.

To perform TUNEL experiments, mouse ovaries were collected and treated overnight at 4℃ in 4% PFA. The tissues were embedded in paraffin wax and sectioned continuously at 7 μm. Apoptotic cells were detected using the TUNEL BrightGreen Apoptosis Detection kit (Vazyme Biotech Co., Ltd, A112). The process of BrdU and TUNEL assays were described in our previous study [68, 74].

### CO-Immunoprecipitation (CO-IP)

The antibodies and beads were combined overnight in a four-degree shaker. Primary ovarian GC samples were collected and lysate in RIPA lysate with PMSF for 15 min, centrifuged at 4000 x g at 4℃ for 15 min. Then the supernatant was combined with beads which bind antibodies and kept overnight at 4℃. The beads were then eluted with the eluents in the Immunoprecipitation Kit (Invitrogen, 10006D). The precipitates were heated in the Sodium Dodecyl Sulfate-Polyacrylamide Gel Electrophoresis (SDS-PAGE) sample buffer and western blotting was performed [75].

### Protein Mass spectrum

The collected primary GC samples (P48) were decomposed by RIPA cracking solution with PMSF for 15 min, centrifuged at 4000 x g for 15 min. Then the supernatant was taken, and then desalted with acetonitrile and dried. Protein levels were determined by Mass Spectrometry Lab of China Agricultural University (Beijing, China) [76].

### Laser-capture microdissection (LCM)-RNA sequencing (Geo-seq)

Fresh ovaries were embedded by optimal cutting temperature compound (OCT, Solarbio, 4583) and continuously sliced at 10 μm using a frozen microtome (Sakura, USA). The slides were then stained with 0.05% crystal violet (Sigma-Aldrich, USA, 548-62-9). Then use the micro cutter (Leica, USA) for cutting the secondary follicle GCs. cDNA library was constructed, purified and sequenced on Illumina HiSeq2000 sequencer according to Picelli et al. ‘s scheme [77]. The DESeq2 R package was then used for differential expression gene (DEGs) analysis.

#### Molecular docking experiment

The predicted structures of USP5, WT1 and P62 were generated by Alphafold. To ensure the accuracy of the docking results, the protein was prepared by the AutoDockTools-1.5.7 [78], and the water molecules were manually eliminated from the protein and the polar hydrogen was added. Docking Web Server (GRAMM) was used for protein-protein docking [79, 80]. The resulting protein-protein complex was also manually optimized by removing water and adding polar hydrogen by the AutoDockTools-1.5.7. Finally, the protein-protein interactions were predicted and the protein-protein interaction figure was generated by PyMOL.

### Statistical analysis

All experiments were repeated at least three times. Results were expressed as the mean ± S.D. Statistical analyses were conducted by GraphPadPrism9 software (GraphPad Software, La Jolla, CA, United States). The statistical significance of the differences between the groups was measured by two-sided ANOVA test. The statistical significances were defined as: *, *P* < 0.05; **, *P* < 0.01; ***, *P* < 0.001, n.s., non-significant.

### Electronic supplementary material

Below is the link to the electronic supplementary material.

Supplementary Material 1

## Data Availability

All data needed to evaluate the conclusions in the paper are present in the paper and the Supplementary Materials.

## Abbreviations

AF: Antral follicle
co-IP: Coimmunoprecipitation
FSH: Follicle-stimulating hormone
FSHR: FSH receptor
GC: Granulosa cell
GO: Gene ontology
H&E: Hematoxylin and eosin
KGN: Human ovarian granulosa cells
LH: Luteinizing hormone
p62/SQSTM1: Sequestosome 1
PF: Primary follicles
PmF: Primordial follicles
PMSG: Pregnant mare serum gonadotropin
SF: Secondary follicle
POR: Poor ovarian response
Ub: Ubiquitination
UPS: Ubiquitin‒proteasome system
USP5: Ubiquitin-specific peptidase 5
WT1: Wilms tumor 1 homolog
