# Supplementary material for: P62 promotes FSH-induced antral follicle formation by directing degradation of ubiquitinated WT1
Source: Cell Mol Life Sci. 2024 May 20;81(1):221. doi: 10.1007/s00018-024-05251-x (PMC11102895; doi:10.1007/s00018-024-05251-x)
Supplement: Supplementary file 1 — Supplementary Material 1 [file 18_2024_5251_MOESM1_ESM.docx]

**Supplementary**


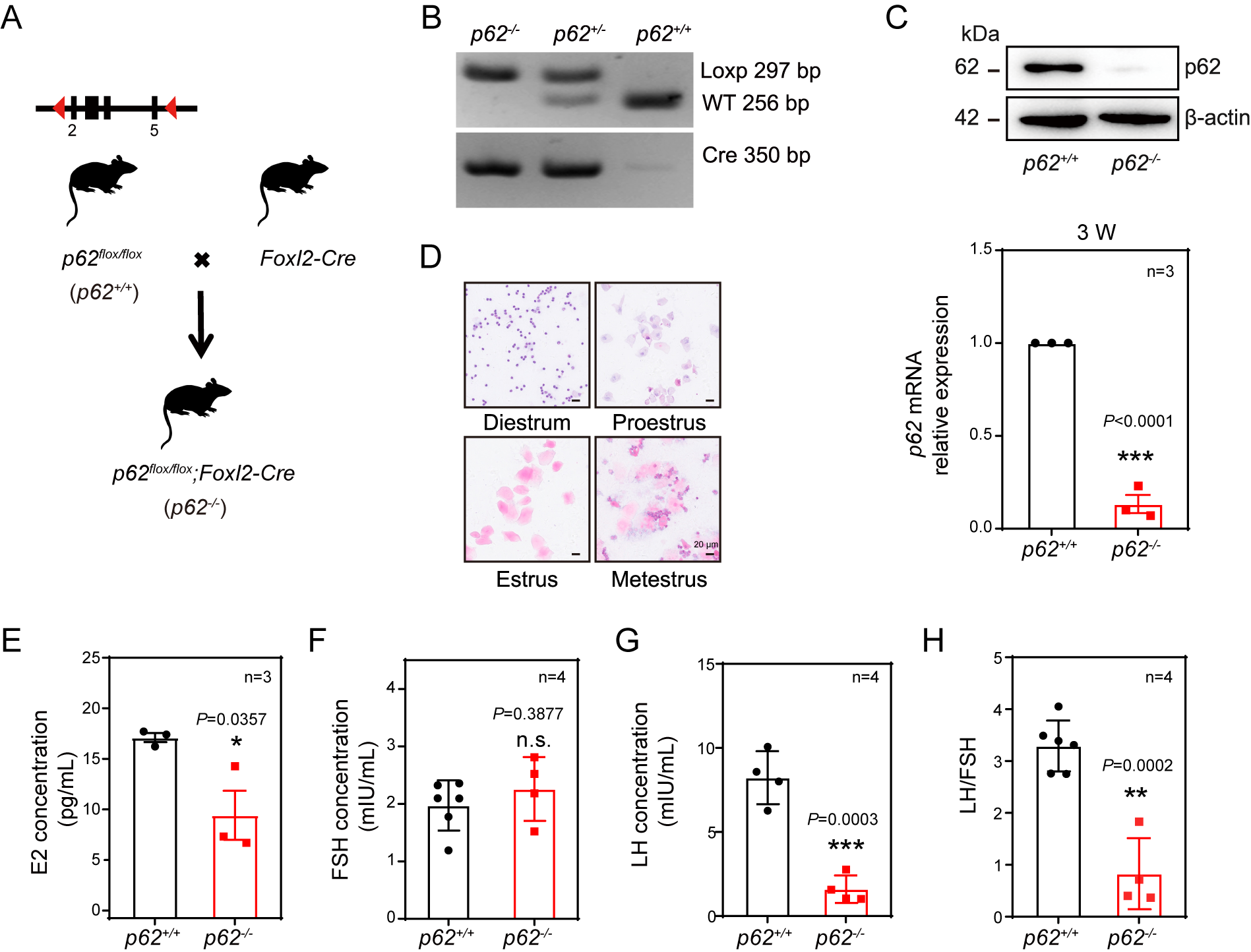


**Fig. S1.** Generation of a *p62^flox/flox^; Foxl2-Cre* (*p62^-/-^*) mouse model and depletion of *p62* impaired female fertility. (A) The strategy of generating a *p62^flox/flox^; Foxl2-Cre* (*p62^-/-^*) mouse model. (B) Schematic diagram of the *p62^flox/flox^* (*p62^+/+^*) and *Foxl2-Cre* alleles. (C) Western blotting and PCR results showed the levels of p62 in the GCs of different genotype mice, revealing the efficient knockout of *p62* in GCs (n=3). (D) An example of identification of the estrous cycle through vaginal smears. Scale bar: 100 μm. (E-H) Serum FSH, LH, E2 content, and LH:FSH ratio of *p62^+/+^* and *p62^-/-^* females at 11 M (n=4).


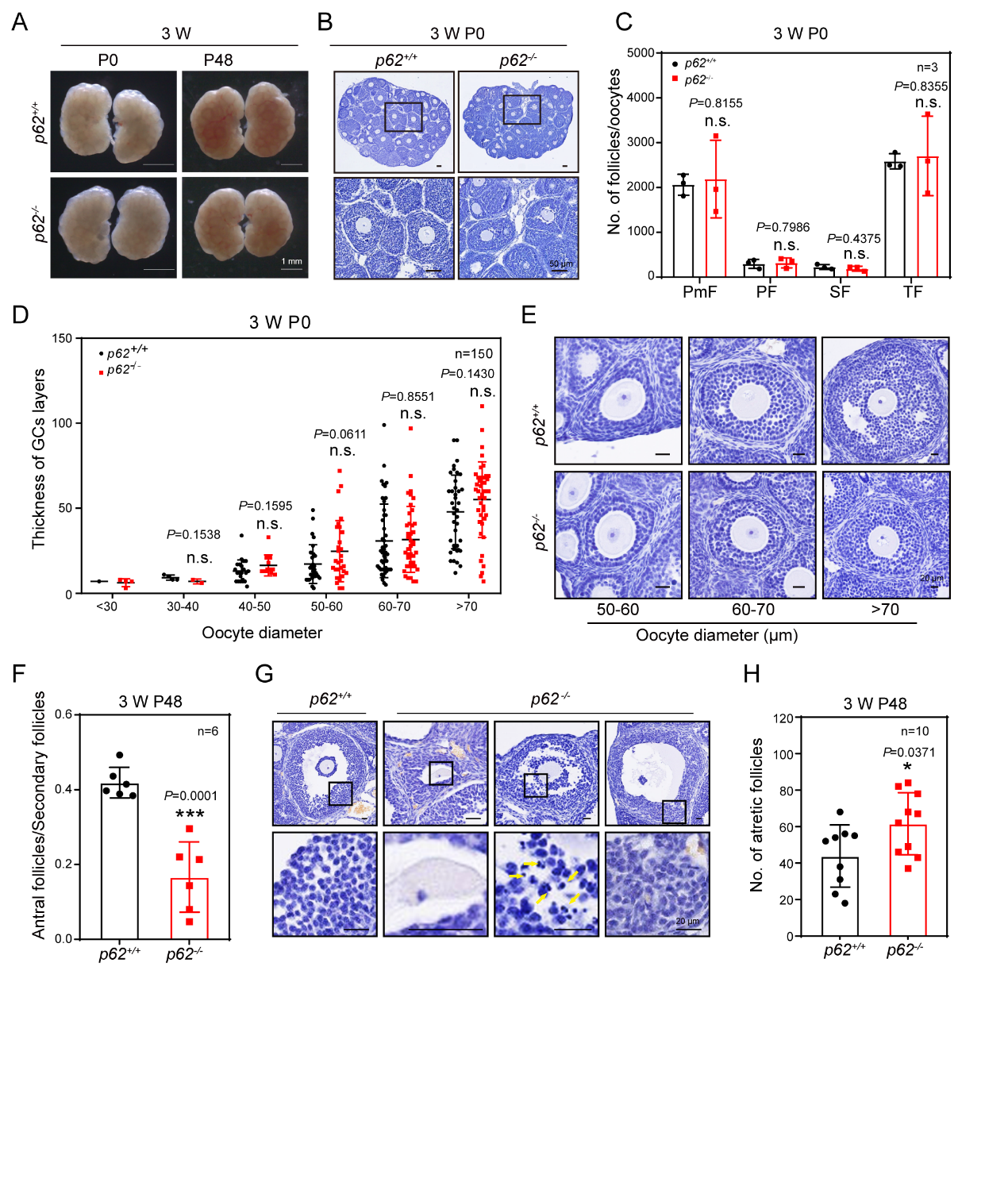


**Fig. S2.** The number of follicles in *p62^+/+^* and *p62^-/-^* mice of three-week-old were like each other. (A) Three weeks old mice were injected with PMSG and the morphology of ovaries at P0 and P48 were evaluated respectively. Scale bar: 1 mm. (B) The hematoxylin-stained ovary sections of P0 mice. The boxed region of each figure was magnified accordingly. Scale bar: 50 μm. (C) Statistics of the numbers of PmFs, PFs, SFs, and TFs in ovaries at P0 (n=3). (D) The thickness of GC layers in all developmental stage follicles of P0 ovaries were compared based on the oocyte diameter (n=150). (E) The follicular morphology in ovaries of *p62^+/+^* and *p62^-/-^* mice were different although the respective oocyte diameters at 50-60 μm, 60-70 μm, and >70 μm were similar between the two groups. Scale bar: 20 μm. (F) Statistics of AFs/SFs (n=6). (G) The morphology of atretic follicles. Scale bar: 20 μm. (H) Statistics of atretic follicles (n=10).


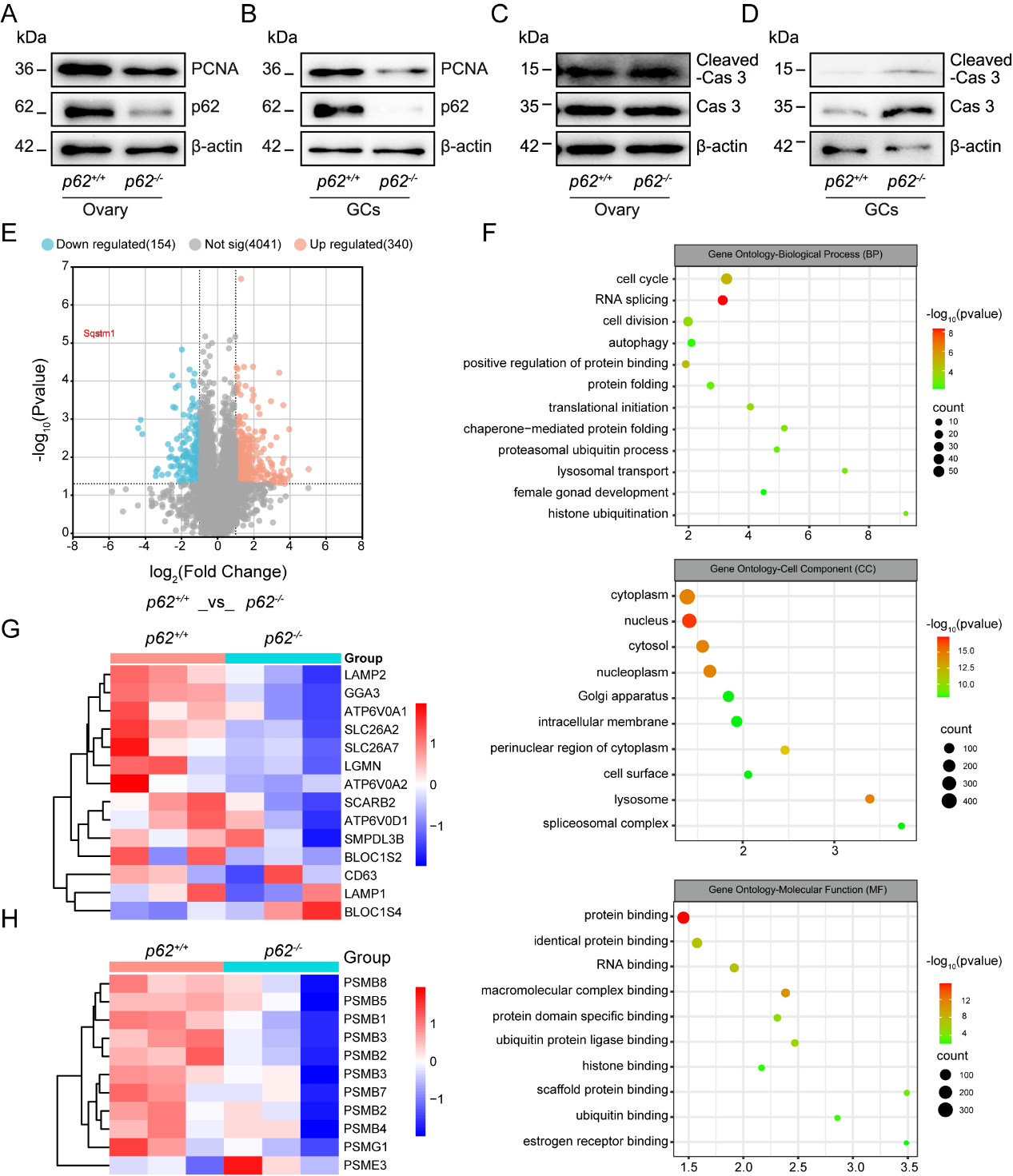


**Fig. S3.** Cell proliferation and apoptosis indicated and mass spectrum data analysis of gene expression pattern in the GCs after mice were treated with PMSG for 48 h. (A and B) Cell proliferation indicated by PCNA-positive GCs in either mice ovaries (A) or GCs (B) (n=3). (C and D) The protein levels of Caspase-3 in both ovaries (C) and GCs (D) after *p62* deletion, respectively (n=3). (E) Totally up-regulated genes (340) and down-regulated genes (154) were obtained by mass spectrogram analysis of three-week-old P48 *p62^+/+^* and *p62^-/-^* mice GCs. (F) Enriched pathways by GO analysis. (G) Heatmap of lysosome-related genes. (H) Heatmap of proteasome-related genes.


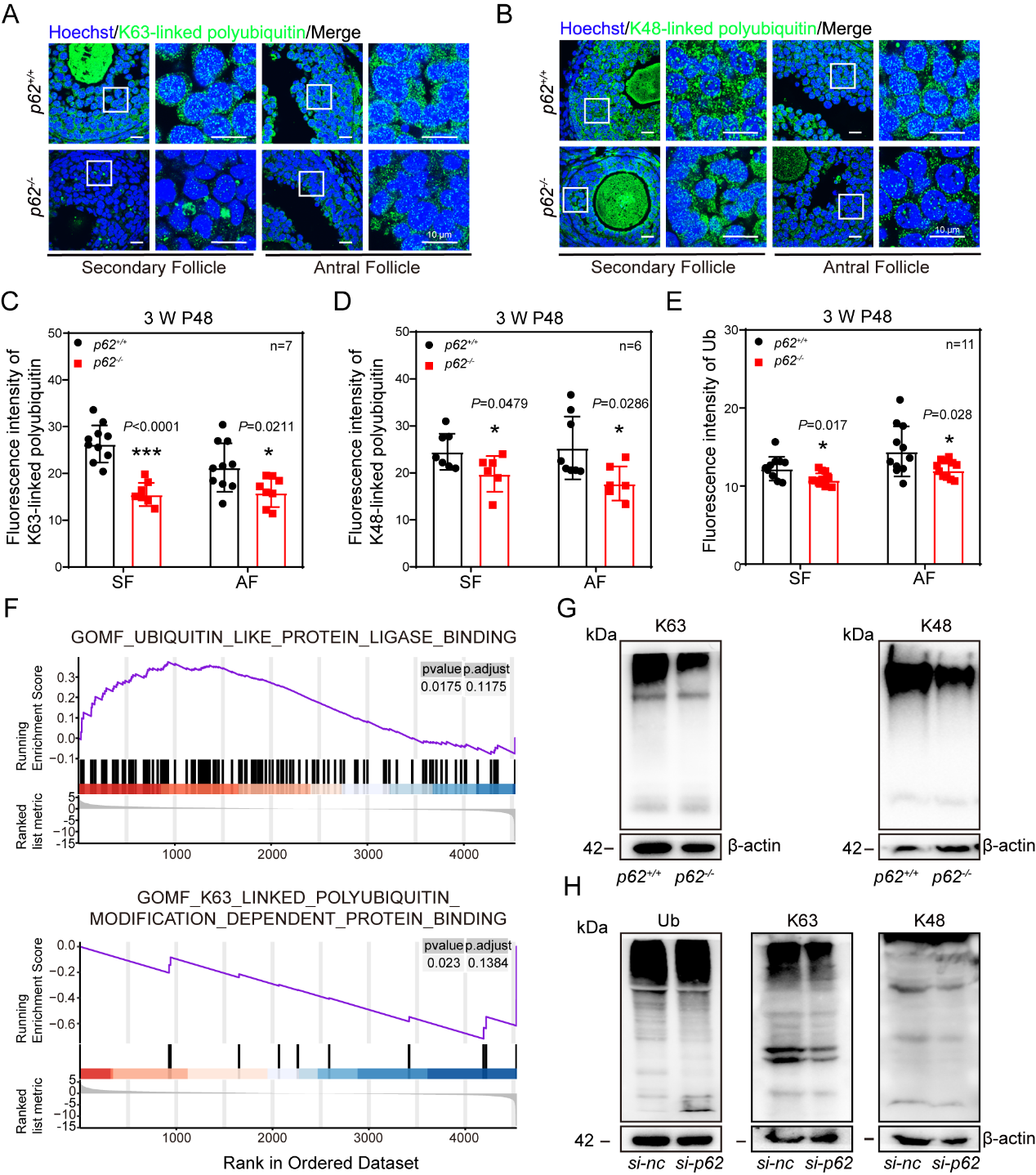


**Fig. S4.** GSEA enrichment and detection of ubiquitination. (A and B) K63-linked (A) and K48-linked (B) polyubiquitin in the GCs indicated by immunofluorescence in three-week-old *p62^+/+^* and *p62^-/-^* mice ovaries. K63-linked and K48-linked polyubiquitin: green; Hoechst: blue. Scale bars: 10 µm. (C-E) Statistics data of K63-linked (C), K48-linked (D) polyubiquitinand and Ubiquitin (E) (n=7, n=6, n=11, Respectively). (F) GSEA analysis of ubiquitin-like protein ligase binding and K63 linked polyubiquitin modification dependent protein binding pathway by mass spectrum. (G) Western blotting for K48-linked and K63-linked polyubiquitin in GCs of *p62^+/+^* and *p62^-/-^* ovaries (n=3). (H) Western blotting for Ub, K48-linked and K63-linked polyubiquitin in KGN of *si-nc* and *si-p62* group (n=3).


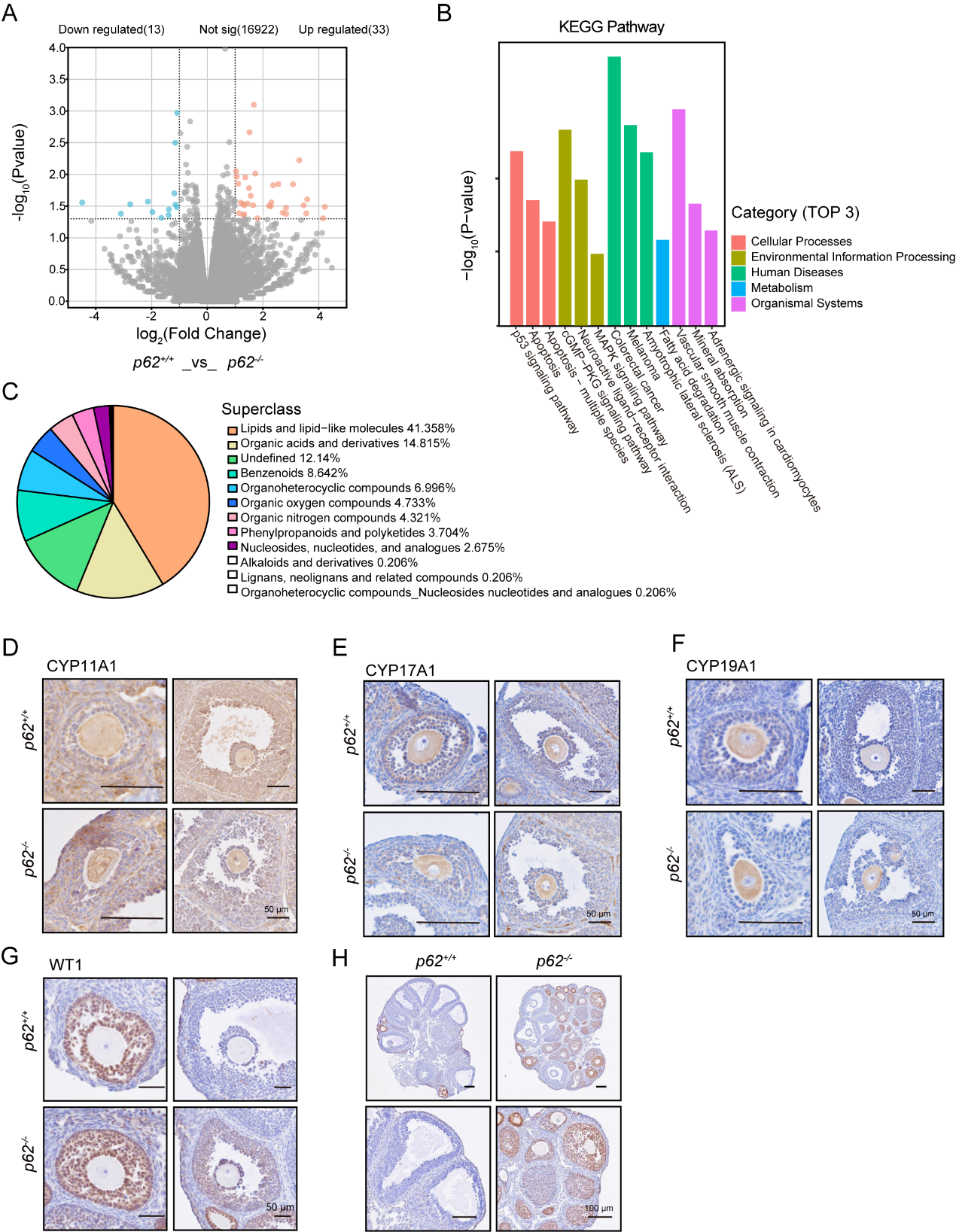


**Fig. S5.** Examination of gene expression after PMSG treatment and GC differentiation-related proteins after depletion of *p62*. (A) Over genes were 33 up-regulated and 13 genes were down-regulated according to RNA-seq analysis of three-week-old mice treated by PMSG for 48 h. (B) KEGG enrichment analysis. (C) Metabolomics analysis in GCs of three-week-old *p62^+/+^* and *p62^-/-^* mice at P48. (D-F) The protein levels of CYP11A1 (D), CYP17A1 (E) and CYP19A1 (F) in GCs of SFs and AFs. Scale bar: 50 μm. (G) Immunohistochemistry detection of WT1 in SFs and AFs. Scale bar: 50 µm. (H) The WT1 protein was shown staining in ovary sections of *p62^+/+^* and *p62^-/-^* mice. Scale bar: 100 µm.


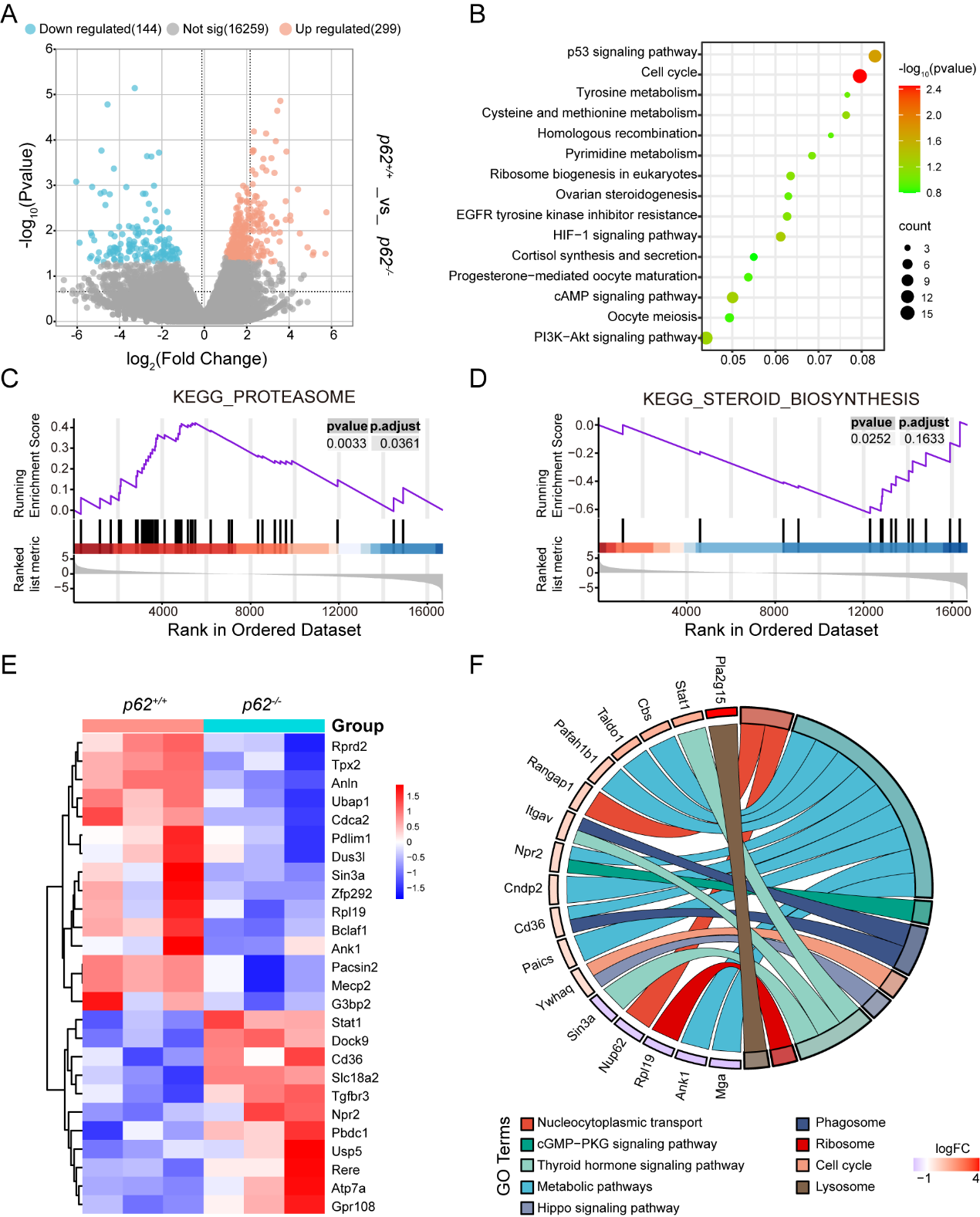


**Fig. S6.** Mass spectrometry data of whole GCs were combined with RNA-seq data of GC-cut parts from the *p62^+/+^* and the *p62^-/-^* mice. (A) RNA-seq analysis of collected GCs from *p62^+/+^* and *p62^-/-^* mice. (B) Enriched pathways by GO analysis in microdissection of GCs. (C-D) GSEA analysis of proteasome (C) and steroid biosynthesis signaling pathways (D). (E) Heatmaps of partial overlapping genes in mass spectra and transcriptomes. (F) Chordal diagram display of GO analysis of partially coincident genes.


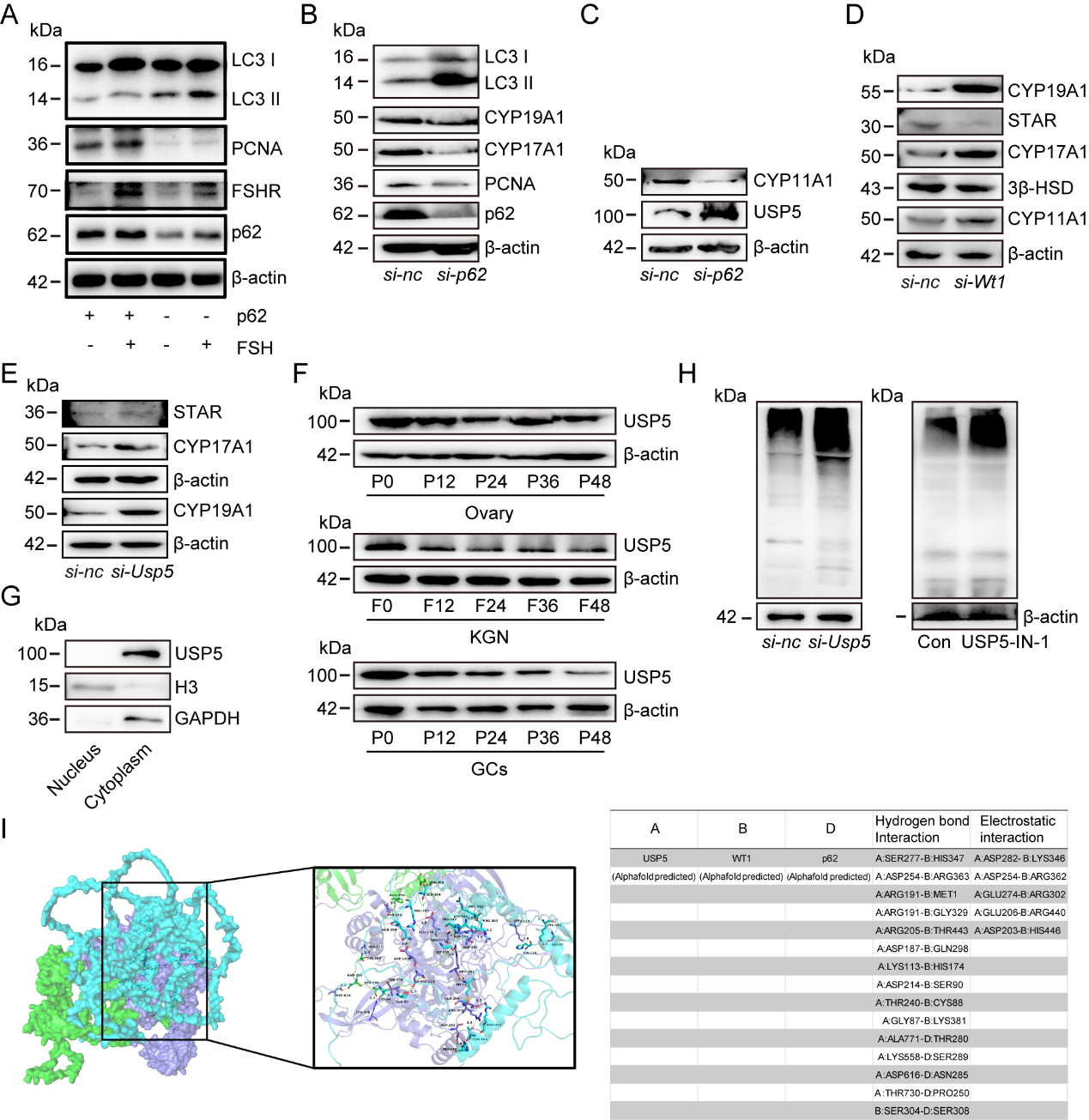


**Fig. S7.** Expression and localization of USP5 and protein detection of KGN cells which treated by *si-p62*, *si-Usp5*, *si-Wt1* and USP5-IN-1. (A) Protein expression of LC3, PCNA, FSHR and p62 in KGN after cells were treated with *si-p62* or *si-nc* in response to FSH. (B and C) Protein expressions of LC3, CYP17A1, CYP19A1, PCNA (B), CYP11A1 and USP5 (C) in KGN cells after *si-p62* treatment. (D) Protein expressions of CYP11A1, CYP17A1, CYP19A1, STAR and 3βHSD in KGN cells of *si-Wt1* and *si-nc*. (E) Protein expressions of CYP17A1, CYP19A1, STAR in KGN cells of *si-Usp5* and *si-nc*. (F) The three-week-old mice were injected with PMSG, and the protein expression of USP5 in the whole ovary and GCs was determined. KGN cells were stimulated by FSH. The protein level of USP5 was determined. (G) USP5 was localized in the cytoplasm of ovarian GCs. (H) Western blotting for ubiquitination in KGN of USP5-IN-1 and *si-Usp5*. (I) The predicted structures of USP5, WT1 and P62 were generated by Alphafold. WT1: blue; p62: green; USP5: purple. The binding sites are shown as the corresponding-colored stick structure. When focusing on the binding region, the binding site is then shown as a presentation of the protein to which it belongs (left). By means of protein-protein interaction analysis in Pymol (right), all functional residues were identified and classified according to their interactions.
